# Multi-mission satellite remote sensing data for improving land hydrological models via data assimilation

**DOI:** 10.1038/s41598-020-75710-5

**Published:** 2020-11-02

**Authors:** M. Khaki, H.-J. Hendricks Franssen, S. C. Han

**Affiliations:** 1 School of Engineering, University of Newcastle, Callaghan, NSW Australia; 2 Agrosphere (IBG-3), Forschungszentrum Jülich GmbH, Jülich, Germany

**Keywords:** Environmental sciences, Hydrology, Computational science

## Abstract

Satellite remote sensing offers valuable tools to study Earth and hydrological processes and improve land surface models. This is essential to improve the quality of model predictions, which are affected by various factors such as erroneous input data, the uncertainty of model forcings, and parameter uncertainties. Abundant datasets from multi-mission satellite remote sensing during recent years have provided an opportunity to improve not only the model estimates but also model parameters through a parameter estimation process. This study utilises multiple datasets from satellite remote sensing including soil moisture from Soil Moisture and Ocean Salinity Mission and Advanced Microwave Scanning Radiometer Earth Observing System, terrestrial water storage from the Gravity Recovery And Climate Experiment, and leaf area index from Advanced Very-High-Resolution Radiometer to estimate model parameters. This is done using the recently proposed assimilation method, unsupervised weak constrained ensemble Kalman filter (UWCEnKF). UWCEnKF applies a dual scheme to separately update the state and parameters using two interactive EnKF filters followed by a water balance constraint enforcement. The performance of multivariate data assimilation is evaluated against various independent data over different time periods over two different basins including the Murray–Darling and Mississippi basins. Results indicate that simultaneous assimilation of multiple satellite products combined with parameter estimation strongly improves model predictions compared with single satellite products and/or state estimation alone. This improvement is achieved not only during the parameter estimation period ($\sim$ 32% groundwater RMSE reduction and soil moisture correlation increase from $\sim$ 0.66 to $\sim$ 0.85) but also during the forecast period ($\sim$ 14% groundwater RMSE reduction and soil moisture correlation increase from $\sim$ 0.69 to $\sim$ 0.78) due to the effective impacts of the approach on both state and parameters.

## Introduction

Studying the terrestrial hydrology is facilitated by developments of land surface models. These models are important to simulate various terrestrial compartments over an extended period of time. Moreover, they are essential for predicting hydrological processes and water storage changes at various temporal and spatial resolutions. The performance of the land surface models, however, can be degraded caused by multiple factors such as uncertainties in model forcings, model parameters, initial and boundary conditions, and simplification of the representation of processes^1,2^. To address this, traditionally additional datasets are integrated with models to improve model estimates.

The data integration approaches have become more popular with the advent of satellite remote sensing. This is related to the satellite’s extensive coverage and high spatial and temporal resolution, especially during the past few decades. Satellite data products can be used to constrain the models, e.g., via data assimilation^3–12^. A number of studies has shown that applying multivariate data assimilation using in-situ and reanalysis estimates^13–19^ could be beneficial. However, despite a few efforts for using multi-mission satellite products for data assimilation^20–22^, the extent of the effectiveness of the approach has not yet been fully investigated^23^. Furthermore, while using the multivariate data assimilation was found to be effective for improving on-line model estimates, its impact on the (long-term) forecasting skill is normally limited if only initial states are updated. The main reason behind this is the important role of the model parameters for simulating fluxes and water storage as well as uncertainty with respect to model forcings (meteorology). Poorly defined parameters, which are not updated during the integration process, can negatively affect the model predictions. These parameters cannot be directly measured thus are difficult to be adjusted. Therefore, a parameter estimation strategy is required to improve the parameters. This is also important to decrease initial state errors in land surface models and consequently enhance forecasts^24^.

In general, the model parameter estimation process adjusts the parameters to increase the consistency between the model simulations and observations based on their uncertainties^25^. In this context, automatic optimization techniques are developed for land surface models, which usually use streamflow observations, especially for rainfall-runoff models^25–28^. There are, however, only a few attempts to tune land surface/water balance model parameters, which are mainly used to model interactions between land (including vegetation) and atmosphere. Many of these efforts have taken advantage of state-of-art satellite remote sensing to improve models^29–34^. Vrugt et al.^35^ proposed a simultaneous optimization and data assimilation method (SODA) to estimate time-invariant parameters while updating state variables. The approach was further applied for operational ensemble streamflow forecasting^36^. Parajka et al.^37^ used soil moisture products from Scatterometer of the European Remote Sensing Satellite (SCAT) to calibrate a conceptual model. Synthetic brightness temperature observations were assimilated by Han et al.^38^ to estimate soil properties as well as soil moisture of the Community Land Model (CLM). In this study, they used the Local Ensemble Transform Kalman Filter (LETKF) to update the augmented state-parameter vector during the assimilation. van Dijk et al.^39^ applied Moderate Resolution Imaging Spectroradiometer (MODIS) data to improve model estimates via the nudging approach and then used the updated estimates dynamically to update parameters. In a different effort, Poovakka et al.^40^ estimated land surface model parameters using both evaporation and soil moisture products.

The main aim of the present study is to apply multi-mission satellite data products to improve a land surface model. This is done by applying an advanced data assimilation method to improve the estimates of different model variables together with model parameters. Terrestrial water storage (TWS) data acquired from the Gravity Recovery And Climate Experiment (GRACE), leaf area index (LAI) from the Advanced Very-High-Resolution Radiometer (AVHRR) instrument, and also soil moisture data from the Soil Moisture and Ocean Salinity (SMOS) as well as Advanced Microwave Scanning Radiometer-Earth Observing System (AMSR-E), are used for the state-parameter estimation. This is the first time that data from multiple satellite missions are used to estimate land surface model parameters using data assimilation. Multivariate data assimilation can be very challenging given various observations with different characteristics and uncertainties, as well as different spatiotemporal resolutions which differ from the model resolution.

Historically, model parameter estimation in combination with data assimilation is either done via an augmented state vector (joint) or a dual approach. The augmented approach has been used often in land hydrological modelling recently^41–47^. Dechant and Moradkhani^48^ applied joint state-parameter estimation using Ensemble Kalman Filter (EnKF) and Particle Filter (PF) and concluded that both methods perform better than only state estimate for improving snow water equivalent predictions. Another effort by Kwon et al.^49^ successfully implemented simultaneous state-parameter estimation in continental-scale radiance assimilation (RA) experiments. Despite these, multiple studies have shown that inconsistency between the estimated parameters and states (e.g., for nonlinear and large-dimensional systems) is an important concern in the joint assimilation method, which can negatively affect the performance of the approach^45,50^. Moradkhani et al.^51^ proposed a dual state-parameter estimation based on sequential Monte Carlo techniques (SMC) for estimating time-variable states and parameters. Khaki et al.^52^ recently proposed an alternative dual data assimilation scheme based on the unsupervised weak constrained ensemble Kalman filter (UWCEnKF). Here, for the first time, we explore the approach to integrate multiple satellite remote sensing data into a model to estimate the model parameters. The experiment is done over two periods, i.e., a parameter estimation and forecast period to better investigate the impact of satellite data integration on the model parameters. The Murray–Darling and Mississippi basins, two large river basin systems, are used as case studies to run and validate the experiment.

## Materials

### Case studies

The two major river basins, Mississippi and Murray–Darling are selected for the experiment given the presence of in-situ measurements to assess the proposed multivariate data assimilation. The Murray–Darling basin is the biggest river system in Australia comprising many wetlands (i.e. more than 30,000) and rivers (i.e. 23), which provide freshwater for, e.g., agriculture, industry, and water use^54^. A large area in the eastern part of the country is covered by the basin, which contains a variety of natural environments, e.g., desert and dry regions (west), rainforest (north), snow covered areas and areas with a larger amount of surface water (south). Historically, the Murray–Darling basin has undergone various extreme droughts and floods. Furthermore, water storage within the basin has also shown large inter-annual and annual variabilities^53,54^. Temperature varies from 0 to $3\,^\circ C$ in the elevated areas in the southeast of the basin in July to 33 to $36\,^\circ C$ for the upper northern parts in January. The same rainfall spatial variability also exists within the Murray–Darling basin, i.e. annual rainfall less than 250 mm in the northwest and excess of 2000 mm in the elevated areas^55^.

Similarly, the Mississippi River basin is an important source of freshwater in North America, which provides water for more than 18 million people and different socioeconomic sectors. Temperature varies strongly within the basin, which leads to large spatial and temporal hydro-climatic variabilities^56,57^. For example, higher temperature ($21\,^\circ C$) along with hot and humid condition exist in May to September while average low temperatures ($-3\,^\circ C$) in January are available in the north caused by various factors such as polar and subtropical jet streams and Arctic cold. Snow line has been progressively migrating northward across the basin^59,60^. Overall, Upper Mississippi areas (e.g., central Minnesota to central Wisconsin) has larger snow cover compared to the other parts of the Mississippi River basin (such as southeast Missouri and southwest Illinois). Showers (and thunderstorms) occur mostly in summers over different parts of the basins while winter precipitation varies from less than 25 mm for the western and northern Great Plains to 75 mm for the Ohio River area and to 130 mm in the south. Climate conditions vary over the different parts of the Mississippi basin and different times of the year^58–60^. This includes semiarid climates in the west, humid condition over the eastern parts, sub-humid climates in the south along with a large discharge rate and multiple flood events across the basin.

In-situ groundwater well data (derived from USGS and the New South Wales Government) are used over both basins to evaluate the estimated groundwater variations from the model with and without data assimilation. To this end, groundwater level data are converted into groundwater storage change values with the help of specific yield values of the basin^2,61–63^. In addition, soil moisture observations from in-situ stations are used to assess the soil moisture estimates at different depths. For this purpose, top, shallow and root-zone soil moisture from the model are compared against in-situ soil moisture measurements at corresponding depths. Over the Mississippi Basin, groundwater and soil moisture measurements are acquired from USGS (https://water.usgs.gov/ogw/data.html) and the International Soil Moisture Network (https://ismn.geo.tuwien.ac.at/), respectively. For the Murray–Darling Basin, the measurements are acquired from New South Wales Government (http://waterinfo.nsw.gov.au/pinneena/gw.shtml) and the moisture-monitoring network^64^.

### Model and data

#### Model

The World-Wide Water Resources Assessment model (W3RA), which was designed and developed by the Commonwealth Scientific and Industrial Research Organisation (CSIRO) is used. W3RA is a Global water balance model, which is distributed on grid basis and simulates water flows and water storage^65^. ERA-interim reanalysis data including meteorological fields of precipitation, maximum and minimum temperature, and downwelling short-wave radiation, are used as model forcings. The model presents the water balance of the soil, groundwater and surface water independently over each grid cell^66^. The water and energy fluxes between the water storages are also modelled for two hydrological response units (HRUs) which occupy different fractions of a grid cell, i.e., tall and deep-root vegetation in HRU1 and short and shallow root vegetation in HRU2. Correspondingly, parameterizations are applied at the sub-grid level^39^. Poovakka et al.^40^ discussed the necessity of calibration for this model, as it is currently limited to a number of catchments where streamflow records and input forcing data are available. The model relies on a variety of parameters such as water holding capacity and effective soil parameters^67^. A detailed list of selected parameters for estimation is presented in Table 1.

These parameters influence mass balance equations underlying the model. Soil albedo and photosynthetic capacity index (PCI) parameters are used to model canopy albedo and outgoing shortwave radiation from the land. Initial retention capacity ($I_0$) and reference event precipitation ($P_{ref}$) are applied to derive surface runoff. These parameters are also connected to rainfall intensity and the soil infiltration distribution. Soil water drainage is estimated based on $\beta$ and field capacity drainage fraction. $F_{ER0}$, $W_{0lim}$ and maximum stomatal conductance ($G_{smax}$) are applied for evaporation modelling, e.g., via rainfall interception evaporation, soil evaporation, and maximum transpiration, respectively. Open water evaporation scaling factor is used to derive open water evaporation, which can have higher uncertainties over large bodies of surface water. Specific leaf area and leaf area index parameters are developed to facilitate vegetation phenology computations^65^.

**Table 1:** W3RA parameters and their associated ranges^65^ used in the parameter estimation process.

Parameter symbol	HRU1 range	HRU2 range	Description
${\alpha }_{dry}$	[0.19–0.35]	[0.19–0.35]	Dry soil albedo (−)
$\beta$	[0.70–8.40]	[0.70–8.40]	Hydraulic conductivity coefficient (−)
*Gsmax*	[0.009–0.05]	[0.009–0.05]	Gsmax-related coefficient (from PCI) (m/s)
$F_{ER0}$	[0.05–0.25]	[0.05–0.25]	coefficient describing rate of wet canopy evaporation to rate of rainfall (−)
$F_{OW}$	[0.60–0.80]	[0.60–0.80]	Open water evaporation scaling factor (−)
$F_{loss,max}$	[0.25–0.50]	[0.25–0.50]	Maximum lose of daytime net radiation (−)
$I_0$	[0–41]	[0–41]	Initial retention capacity (mm)
${\wedge }_{ref}$	[1.30–3.50]	[1.30–2.50]	Reference LAI determining canopy cover (−)
$P_{ref}$	[54–1000000]	[254–1000000]	Reference event precipitation (to generate runoff) (mm/d)
$C_{SLA}$	[0.70–71]	[0.70–71]	Specific leaf area coefficient (m$^2$/kg)
*PCI*	[0.01–1.00]	[0.01–1.00]	Photosynthetic capacity index (−)
$W_{0lim}$	[0.60–0.89]	[0.60–0.89]	Water content at top soil (where evaporation is decreased) (−)

#### Satellite remote sensing

Three main satellite products are used for data assimilation to update states and estimate parameters. TWS changes are derived from level 2 (L2) GRACE products (up to degree and order 90). L2 coefficients and their associated full error covariance information are acquired from the ITSG-Grace2014 gravity field model^68^. Post-processing steps are done following Khaki et al.^69^ and Khaki and Awange^70^ to calculate TWS changes between 2003 and 2016. The data is then used to update the summation of different water storage components from the model including groundwater, different soil layers, and surface water storage (see details in “Methodology” section). TWS error covariances (to be used in data assimilation) are computed from potential coefficients following Schumacher et al.^46^.

The National Oceanic and Atmospheric Administration (NOAA) of LAI Climate Data Record (CDR; version 4) and Fraction of Absorbed Photosynthetically Active Radiation (FAPAR)^71^ are obtained for the period of 2003–2016. The data were produced by the University of Maryland and the NASA Goddard Space Flight Center (GSFC) on a daily $0.05^\circ \times 0.05^\circ$ global scale. LAI products are used for data assimilation given their potential to improve modelling skills^72^. LAI has a major impact on estimating evapotranspiration (ET) and precipitation interception, thus, can be very useful for data assimilation^71^. Following Fox et al.^73^, a constant error standard deviation of 0.2 (m$^2$ m$^{-2}$) is assumed for the LAI from satellite.

Soil moisture products are achieved over the same period, i.e., 2003–2011 from AMSR-E (Level-3)^74^ and 2011–2016 from SMOS (Level 3 Centre Aval de Traitement des Donnees SMOS)^75^. These data are used during assimilation to control the model surface soil moisture content. Regarding soil moisture measurement uncertainty, we followed Leroux et al.^76^ and De Jeu et al.^77^ and assumed 0.04 (m$^3$ m$^{-3}$) error for SMOS and 0.05 (m$^3$ m$^{-3}$) error for AMSR-E observations.

#### Water fluxes

Additional datasets of precipitation, total evapotranspiration, and water discharge are used to constrain the water balance through in the UWCEnKF implementation (see details in “Data assimilation” section). These data are derived from Khaki et al.^69^, in which data from different sources, e.g., satellite, reanalysis, and gauge-based measurements (from multiple sources over the Mississippi and Murray–Darling basins), are merged to achieve the best estimates over different basins. Note that the datasets applied here for water budget constraint are mostly independent from those applied for running the model except for precipitation, e.g., the Tropical Rainfall Measuring Mission (TRMM) is used in both ERA-interim forcing and the merged precipitation product for the water budget constraint. Nevertheless, this dependency between the products is not a limitation for our data assimilation experiments as it was shown that the water budget closure, where the water flux observations are used is not affected by this^69,78^.

## Methodology

### Sensitivity analysis

A sensitivity analysis following Cannavo^79^ is carried out to measure the model response to parameter changes. This is done to identify the parameters that significantly affect the model output. The analysis will also increase our understanding of the impact of model parameters on model simulations. The selected approach here is a global sensitivity analysis that contrary to so-called local sensitivity analysis assesses sensitivity over the entire input parameter space. It is a variance-based method that investigates the contribution of each input parameter to the total variance of the output, i.e., $\mathbf{y} = f(\mathbf{X})$ and $\mathbf{X} = (x_1,x_2, \ldots , x_n)$ with $\mathbf{n}$ being the number of input parameters ($\mathbf{x}$). The objective is to measure the importance of input on the variance of the output, namely the sensitivity ($S_i$) of $\mathbf{y}$ to $x_i$ through,

1 $$ S_i = \frac{var\{E[\mathbf{y}|x_i]\}}{var\{\mathbf{y}\}}. $$

This is known as the first order sensitivity index by Sobol^80^. Analytical solution of Eq. 1 for a non-linear high dimensional system is not possible, thus, a numerical approximation is needed. This can be facilitated using the Fourier Amplitude Sensitivity Test (FAST) and the Monte Carlo algorithm for numerical approximation. Here we apply the latter following Cannavo^79^, where a sequence of random points of length *N* can be used to approximate the solution for $N \,\rightarrow \, \infty$. This allows for evaluating a multidimensional integral using a Monte-Carlo technique. Consider two uniformly distributed independent random points *A* and *B* (with a size of $N \times n$). $A=[{\alpha }^A,{\beta }^A]$ and $B=[{\alpha }^B,{\beta }^B]$ are composed of *N* trial sets for the evaluation of $\mathbf{y}$. The model ($f(\mathbf{X})$) can be evaluated in these two points: *f*(*A*) and $f({\alpha }^A,{\beta }^B)$. Using this method, the influence of different variables and their subsets on the model can be analyzed. In practice, this method draws *A* and *B* and form *C* ($C_i, i=1, \ldots , n$) in a way that its $i{th}$ column is equal to the $i{th}$ column of *B*, and its remaining columns are from *A*. Using these sample inputs, the model is run to derive corresponding model evaluations ($f(A), f(B), f(C_i)$). These are then used to calculate the sensitivity indices using,

2 $$ var\{E[\mathbf{y}|x_i]\} = \frac{1}{N} \displaystyle \sum _{j=1}^{N} {f(B)}_j [{f(C_i)}_j-{f(A)}_j]. $$

Using this method one can measure the sensitivity of the model to a given parameter based on its contribution to the variance of the model output see more details in^79^.

### Data assimilation

#### UWCEnKF

The main aim of UWCEnKF is to update the system state and its parameters in a dual way while accounting for water balance when incorporating new observations. Here, we present a summary of the approach and more details can be found in Khaki et al.^52^. Ait-El-Fquih et al.^45^ proposed a new dual EnKF scheme following the one-step-ahead (OSA) smoothing and showed that this could improve data assimilation performance by imposing more information to the system. Their approach comprises two interactive EnKF filters for state-parameter estimation. Khaki et al.^52^ extended this to a water balance system by enforcing an additional constraint. The approach includes different steps; it first uses the state forecast ensemble to update the parameters through EnKF-like update, as well as to compute the OSA smoothing ensemble. The updated parameters and state variables are then integrated with the model to obtain the next state forecast ensemble in the second EnKF, which will be used to acquire the analysis ensemble. Despite the addition of the second EnKF implementation compared to the traditional dual-EnKF due to the OSA smoothing part, it has been shown that this only increases the computational cost minimally while it considerably enhances the performance of the dual approach^44,45,52^. For the state-parameter estimation problem in a discrete-time dynamical system, one can write,

3 $$ \left\{ \!\! \begin{array}{l} \mathbf{x}_t = {{{\mathcal {M}}}}_{t-1}(\mathbf{x}_{t-1},\mathbf{\theta }_{t-1}) + \mathbf{\nu }_{t-1}, \\ \mathbf{y}_t = \mathbf{H}_t \mathbf{x}_t+ \mathbf{w}_t, \end{array} \right. $$

where $\mathbf{x}_t \in {\mathbb {R}}^{n_x}$ is the system state vector (with dimension $n_x$) and $\mathbf{y}_t \in {\mathbb {R}}^{n_y}$ is the observation vector (with dimension $n_y$) at time *t*. $\theta \in {\mathbb {R}}^{n_\mathbf{\theta }}$ represents the parameter vector of dimension $n_\mathbf{\theta }$. In Eq. (3), the model operator is indicated by ${{{\mathcal {M}}}}_{t-1}(.)$, which is used to forward the state vector from $t-1$ to *t*, and the observational (design) operator at time *t* is shown by $\mathbf{H}_t$. The model and observation process noises are represented by $\mathbf{\nu }_{t-1} \sim {{\mathcal {N}}}(\mathbf{0} , \mathbf{Q}_t)$ and $\mathbf{w}_t \sim {{\mathcal {N}}}(\mathbf{0} , \mathbf{R}_t)$, respectively, with state covariance matrix $\mathbf{Q}_t$ and observation covariance matrix $\mathbf{R}_t$. To solve Eq. (3), UWCEnKF applies a dual EnKF scheme comprising two interactive EnKF filters for state-parameter estimation. Each step of the filter is presented below.

- *Parameter estimation.* Starting from $\{\mathbf{x}_{t-1}^{a,(i)},\mathbf{{\theta }}_{t-1}^{(i)}\}_{i=1}^n$ (with *n* being the ensemble number and *a* standing for analysis step), the process begins with integrating state and parameters within the model to derive forecast state ($\tilde{\mathbf{x}}_t^{f,(i)}$) as,
  4 $$ \left\{ \!\! \begin{array}{l} \tilde{\mathbf{x}}_t^{f,(i)} = {{\mathcal {M}}}_{t-1}(\mathbf{x}_{t-1}^{a,(i)},\mathbf{\theta }_{t-1}^{(i)}) + \mathbf{\nu }_{t-1}^{(i)}, \\ \tilde{\mathbf{y}}_t^{f,(i)} = \mathbf{H}_t \tilde{\mathbf{x}}_t^{f,(i)}+ \mathbf{w}_t^{(i)}. \end{array} \right. $$
  The observation forecast $\{ \tilde{\mathbf{y}}_t^{f,(i)}\}_{i=1}^n$ is then used to calculate the analysis parameter ensemble $\{\mathbf{{\theta }}_{t}^{(i)}\}_{i=1}^n$ by,
  5 $$ \mathbf{\theta }_{t}^{(i)} = \mathbf{\theta }_{t-1}^{(i)}+ \mathbf{P}_{\mathbf{\theta }_{t-1}, \tilde{\mathbf{x}}_t^{f}} \mathbf{H}^T \underbrace{ [\mathbf{H} \mathbf{P}_{\tilde{\mathbf{x}}_t^{f}} \mathbf{H}^T + \mathbf{R}_t]^{-1} [\mathbf{y}_t - \tilde{\mathbf{y}}_t^{f,(i)}] }_{\varvec{\mu }_t^{(i)}}, $$
  with the sample forecast error covariance matrix $\mathbf{P}_{\mathbf{x}_t^f}$ and the sample cross-covariance matrix between the previous parameter vector and current forecast errors $\mathbf{P}_{\mathbf{\theta }_{t-1}, \tilde{\mathbf{x}}_t^{f}}$,
  6 $$ \mathbf{P}_{\tilde{\mathbf{x}}_t^{f}} = (n-1)^{-1} \mathbf{S}_{\tilde{\mathbf{x}}_t^{f}} \mathbf{S}_{\tilde{\mathbf{x}}_t^{f}}^T , $$
  7 $$ \mathbf{P}_{\mathbf{\theta }_{t-1}, \tilde{\mathbf{x}}_t^{f}} = (n-1)^{-1} \mathbf{S}_{\mathbf{\theta }_{t-1}} \mathbf{S}_{\tilde{\mathbf{x}}_t^f}^T, $$
  where $\mathbf{S}$ is ensemble perturbation and can be calculated as a difference between ensemble members and ensemble mean.
- *State estimation.* Traditionally, the analysis parameters are used to recalculate the forecast ensemble in the standard dual EnKF by integrating $\{ \mathbf{x}_{t-1}^{a,(i)}\}_{i=1}^n$ into the model based on the updated parameters. Ait-El-Fquih et al.^45^ showed that the implementation of the OSA smoothing step, which is a measurement update based on the current observation can lead to a better state estimate. The smoothing state $\{ \mathbf{x}_{t-1}^{s,(i)}\}_{i=1}^n$ can be derived according,
  8 $$ \mathbf{x}_{t-1}^{s,(i)} = \mathbf{x}_{t-1}^{a,(i)} + \mathbf{P}_{\mathbf{x}_{t-1}^a, \tilde{\mathbf{x}}_t^{f}} \mathbf{H}^T \varvec{\mu }_t^{(i)}, $$
  9 $$ \mathbf{P}_{\mathbf{x}_{t-1}^a , \tilde{\mathbf{x}}_t^{f}} = (n-1)^{-1} \mathbf{S}_{\mathbf{x}_{t-1}^a} \mathbf{S}_{\tilde{\mathbf{x}}_t^f}^T, $$
  with $\mathbf{P}_{\mathbf{x}_{t-1}^a , \tilde{\mathbf{x}}_t^{f}}$ being the sample cross-covariance matrix, calculated from the analysis states at $t-1$ and forecast states at *t*. Next, similar to the standard EnKF, the forecast step is applied but using the updated parameters to forward states in time (from $t-1$ to *t*). This is done using $\{\mathbf{x}_{t-1}^{s,(i)}, \mathbf{\theta }_{t}^{(i)}\}_{i=1}^n$ via,
  10 $$ \left\{ \!\! \begin{array}{l} \mathbf{x}_t^{f,(i)} = {{\mathcal {M}}}_{t-1}(\mathbf{x}_{t-1}^{s,(i)},\mathbf{\theta }_{t}^{(i)}) + \mathbf{\nu }_{t-1}^{(i)}, \\ \mathbf{y}_t^{f,(i)} = \mathbf{H}_t \mathbf{x}_t^{f,(i)}+ \mathbf{w}_t^{(i)}. \end{array} \right. $$
  Next, the state vector is to be updated based on the observation vector. This step is implemented to calculate the state analysis $\{ \tilde{\mathbf{x}}_{t}^{a,(i)}\}_{i=1}^n$ by:
  11 $$ \tilde{\mathbf{x}}_t^{a,(i)} = \mathbf{x}_t^{f,(i)} + \mathbf{P}_{\mathbf{x}_t^f,\mathbf{y}_t^f}{} \mathbf{P}_{\mathbf{y}_t^f}^{-1} [\mathbf{y}_t - \mathbf{y}_t^{f,(i)}]. $$
  Khaki et al.^78^ and Khaki et al.^69^ showed that assimilation of observations related to the storage term, especially from GRACE TWS can break the balance between water flux components, namely water storage change ($\Delta \mathbf{s}$), precipitation ($\mathbf{p}$), evaporation ($\mathbf{e}$), and water discharge ($\mathbf{q}$) in the water balance equation, which can be formulated as,
  12 $$ \mathbf{d}_t = - \mathbf{x}_t+ \mathbf{x}_{t-1}+ \mathbf{p}_t- \mathbf{e}_t- \mathbf{q}_t+ {\varvec{\xi }}_t, $$
  with noise ${\varvec{\xi }}_t$ and corresponding covariance ${{\varvec{\Sigma }}}_t$ associated with the flux observations. To account for the imbalance issue, Khaki et al.^78^ imposed a constraint based on the water balance equation and using an EnKF-like update. To do this, a correction is applied based on the estimated imbalance as,
  13 $$ \mathbf{z}_t = \mathbf{G} \mathbf{x}_t + \mathbf{L} \mathbf{x}_{t-1} + {\varvec{\xi }}_t, $$
  where $\mathbf{z}_t {\mathop {=}\limits ^{\rm def}} \mathbf{d}_t- \mathbf{p}_t+ \mathbf{e}_t+ \mathbf{q}_t$ is introduced as “pseudo-observation”. In this equation, $\mathbf{L}$ is an $n_z \times n_x$ identity matrix, and $\mathbf{G} = - \mathbf{L}$ (here, $n_z = n_x$). Contrary to a standard EnKF that only computes states in the analysis step, UWCEnKF estimates pseudo-observation noise covariance along with the states. This leads to the computation of constrained states from unconstrained state analysis ($\{ \tilde{\mathbf{x}}_{t}^{a,(i)}\}_{i=1}^n$) in a second analysis step. A recursive algorithm exists in UWCEnKF to efficiently compute the analysis state $\{\mathbf{x}_{t}^{a,(i)}\}_{i=1}^n$ based on the pseudo-observation noise covariance matrix (${\hat{{\varvec{\Sigma }}}}_t$). The second update step, thus, involves cyclic iterations to adjust the analysis state for $\ell = 0 \ldots L$ (with *L* being the iteration number) as,
  14 $$ \mathbf{z}_t^{f,(i,\ell )} = \mathbf{G}\tilde{\mathbf{x}}_t^{a,(i)}+ \mathbf{L} \tilde{\mathbf{x}}_{t-1}^{s,(i)} + \varvec{\xi }_t^{(i,\ell )}; \quad \varvec{\xi }_t^{(i,\ell )} \sim {{\mathcal {N}}}(\mathbf{0} , {\hat{{\varvec{\Sigma }}}}_t^{(\ell )}), \quad i=1, \ldots , n, $$
  15 $$ \mathbf{x}_t^{a,(i,\ell )} = \tilde{\mathbf{x}}_t^{a,(i)} + \mathbf{P}_{\tilde{\mathbf{x}}_t^a, \mathbf{z}_t^{f,\ell }} \underbrace{ [\mathbf{M} \mathbf{P}_{\varvec{\eta }_t} \mathbf{M}^T + {\hat{{\varvec{\Sigma }}}}_t^{(\ell )} ]^{-1} [\mathbf{z}_t - \mathbf{z}_t^{f,(i,\ell )}]}_{\nu _t^{(i,\ell )}} , \quad i =1, \ldots , n, $$
  16 $$ \mathbf{x}_{t-1}^{s,(i,\ell )} = \tilde{\mathbf{x}}_{t-1}^{s,(i)} + \mathbf{P}_{\tilde{\mathbf{x}}_{t-1}^s, \mathbf{z}_t^{f,\ell }} \times \nu _t^{(i,\ell )}, \quad i =1, \ldots , n, $$
  with $\mathbf{M} {\mathop {=}\limits ^{\rm def}} [\mathbf{G} , \mathbf{L}]$ and sample covariances $\mathbf{P}_{\tilde{\mathbf{x}}_t^a , \mathbf{z}_t^{f,\ell }}$ and $\mathbf{P}_{\tilde{\mathbf{x}}_{t-1}^s , \mathbf{z}_t^{f,\ell }}$ that are derived from ${\{ \tilde{\mathbf{x}}_t^{a,(i)} \}}_{i=1}^n$, ${\{ \tilde{\mathbf{x}}_{t-1}^{s,(i)} \}}_{i=1}^n$ and ${\{ \mathbf{z}_t^{f,(i,\ell )} \}}_{i=1}^n$. $\mathbf{P}_{{\varvec{\eta }}_t}$ can also be calculated from ${\{\varvec{\eta }_t^{(i)}\}}_{i=1}^n$ with $\varvec{\eta }_t^{(i)} {\mathop {=}\limits ^{\rm def}} [(\tilde{\mathbf{x}}_{t}^{a,(i)})^{T} , (\tilde{\mathbf{x}}_{t-1}^{s,(i)})^{T} ]^{T}$. At each iteration ${\hat{{\varvec{\Sigma }}}}_t^{(\ell )}$ is computed from the new state and it is then used again in Eqs. (14)–(16)^12^.

#### Data assimilation setup

An experiment is designed to monitor the performance of multivariate data assimilation. The study period is divided into three parts: 2000–2002 to generate the initial ensemble, 2003–2012 to assimilate observations and estimate model parameters (i.e. assimilation periods), and 2013–2016 to investigate the impact of the estimated parameters on model simulations in the absence of assimilation (i.e. forecasting period). The spin-up is made for $m=30$ ensemble members for the period 2000–2002. This is done by perturbing the meteorological forcing fields, i.e. for precipitation: $\times {{\mathcal {N}}}(\mathbf{0} , 0.3)$, for shortwave radiation: $+ {{\mathcal {N}}}(\mathbf{0} , 50)$, and for temperature: $+ {{\mathcal {N}}}(\mathbf{0} , 2)$. Model errors are mainly caused by errors in the initial condition, forcing data, and model parameters. The above perturbation process accounts for the first two error sources while the model structure error is not considered here. Nevertheless, ensemble inflation applied in the assimilation process (explained below) allows the ensemble to largely account for this error^81,82^. A parameter ensemble is produced by drawing (30) random samples from each parameter’s HRU defined range (cf. Table 1). The state vector for data assimilation includes soil moisture at three layers of top (up to 7–9 cm soil layer), shallow (up to 30 cm soil layer), and deep-zone layers (up to 100 cm soil layer), surface and snow water storage, groundwater and LAI. The observation vector contains GRACE TWS observations, satellite soil moisture, and LAI products. Cumulative distribution function (CDF) matching is used for rescaling the observations (TWS, soil moisture, and LAI) to match those from the model^22,83^.

The observational operator ($\mathbf{H}_t$) converts the state variables into the observation space by taking into account discrepancies between the model and observation spatial resolutions. It aggregates model state variables at multiple grid cells to $1^\circ$ to be updated by $1^\circ$ GRACE TWS data. Top layer soil moisture variables at every $0.25^\circ$ are updated by satellite soil moisture measurements (i.e. $0.25^\circ$ AMSR-E and SMOS). LAI observations are spatially averaged and assimilated at the same resolution as of model ($0.125^\circ$). To deal with the observations different temporal resolution, all observations are rescaled to the monthly scale (same as GRACE products) and assimilated on a monthly basis. This scale is also selected because it allows easier water budget constraint implementation provided in the second step of UWCEnKF, where water balance equation is applied using TWS changes over consecutive months. The monthly corrections as a result of data assimilation are added as offsets to the state vectors at the last day of each month to generate the ensembles for the next month assimilation step^2,84^.

To enhance EnKF performance during assimilation ensemble inflation and localization are applied. It has been shown by literature^85,86^ that ensemble-based data assimilation methods are sensitive to the size of ensemble. Generally, a larger number of ensemble members can better span the state-observation space and lead to better results but at the expense of strongly increased computation needs. To address this, ensemble inflation and localization methods are usually used to tackle filter divergent or inaccurate estimation^87^ for a small ensemble size and to avoid filter inbreeding. Ensemble inflation increases ensemble deviation from the ensemble-mean by applying a small coefficient ($[1.1-1.3]$ for the parameter and state updates) to ensemble members^88^. Localization using the Local Analysis (LA) scheme is also applied. It performs by spatially limiting the assimilation process within a certain distance from a grid point^10,89^. The suggested values ($3^\circ$) by Khaki et al.^10^ are used as localization radii to achieve the best outcomes using a trial and error.

As mentioned, the experiment is undertaken over the Murray–Darling and Mississippi basins given good data availability. To assess the results, model simulations are spatially interpolated to the nearest in-situ stations (cf. “Case studies” section). Once the simulation time series are generated at these locations, three evaluation metrics including standard deviation (STD), Root-Mean-Squared Error (RMSE), and correlation values are calculated with respect to the independent in-situ measurements. RMSE and STD are particularly useful to respectively investigate the distance between the simulations and in-situ measurements and the spread of simulations around the mean. These show how accurate and precise the results are. Note that only time series anomalies (i.e. time series minus their temporal average) are used for the validation. To better investigate the performance of multivariate data assimilation for state-parameter estimation, its results are compared with the open-loop (model run without assimilation), multivariate data assimilation without parameter estimation, and univariate data assimilations cases, where only one of the remote sensing products (GRACE TWS, soil moisture and LAI observations) is used for state-parameter estimation. For convenience, hereafter the assimilation with parameter estimation approach will be called A/Par and the assimilation only method without parameter estimation will be called A/O.

## Results

### Sensitivity analysis

**Figure 1:**
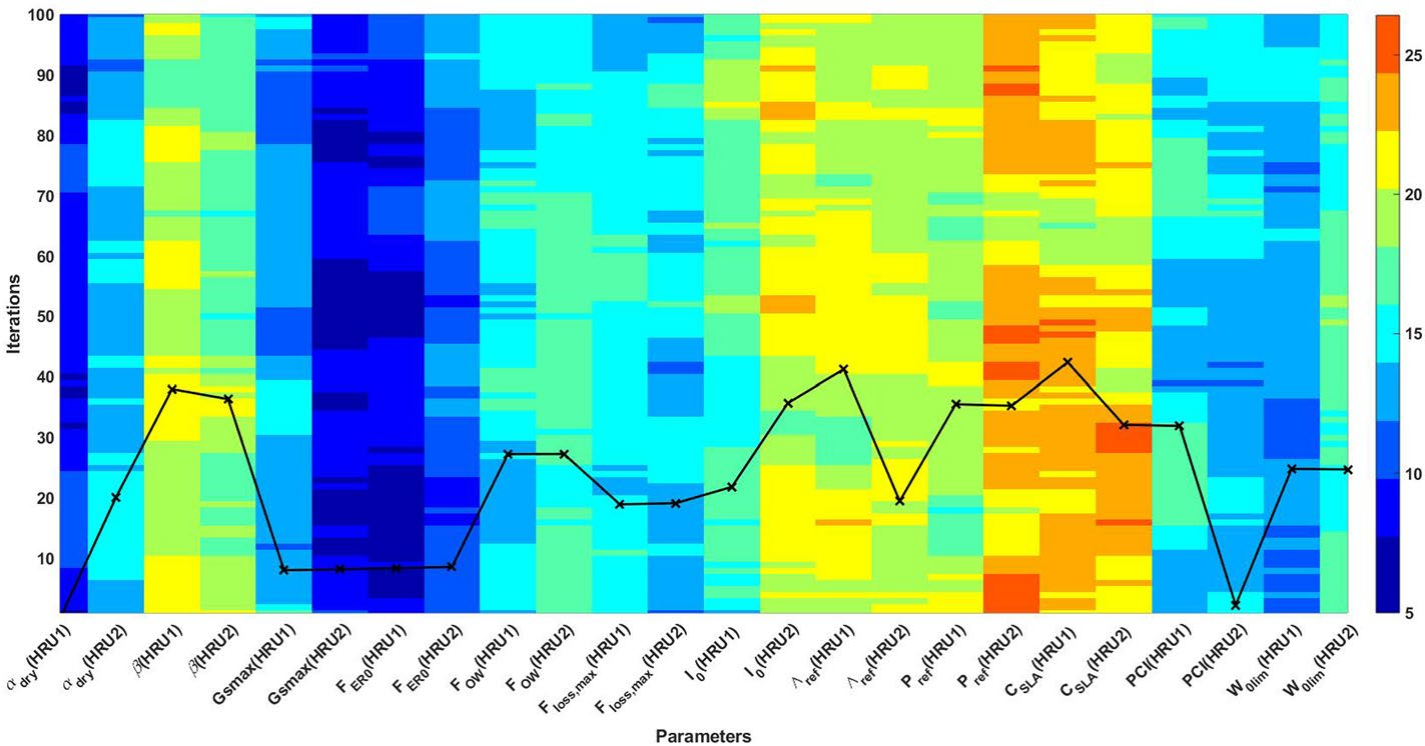
The results of the sensitivity analysis from 100 different samples. The relative weights of different parameters (the average over the 100 iterations scaled by the factor 2 for a better presentation) is also plotted in the figure with a solid black line.

The results of the sensitivity analysis are shown in Fig. 1. Estimated sensitivity weights of parameters (cf. Table 1) for each iteration of total 100 different iterations (using 100 different sets of matrices, see “Sensitivity analysis” section) are spatially averaged to show the relative weights of the model parameters and their influence on model output. In addition, the average parameter weights (for 100 iterations) are also plotted in the figure by a solid black line. It can be seen that larger weights are assigned to a group of parameters including $C_{SLA}$, $\Lambda _{ref}$, $I_{0}$ $P_{ref}$, and $\beta$ with $C_{SLA}$ and $\Lambda _{ref}$ having the biggest weights amongst all parameters. This can show the fundamental impact of specific leaf area and its interaction with light and moisture (humidity) levels within the study area. These larger weights corresponding to more model sensitivity can be observed over a majority of iterations. Some of the other parameters such as $G_{smax}$ and $F_{ER0}$ represent less impact on the model outputs. From Fig. 1, it can be seen that sensitivity of parameters (e.g., *PCI* and ${\alpha }_{dry}$) differ between HRU’s. These indicate the effect of the model parameter variations on the simulation results, which highlights the importance of an accurate selection of parameters for estimation.

**Figure 2:**
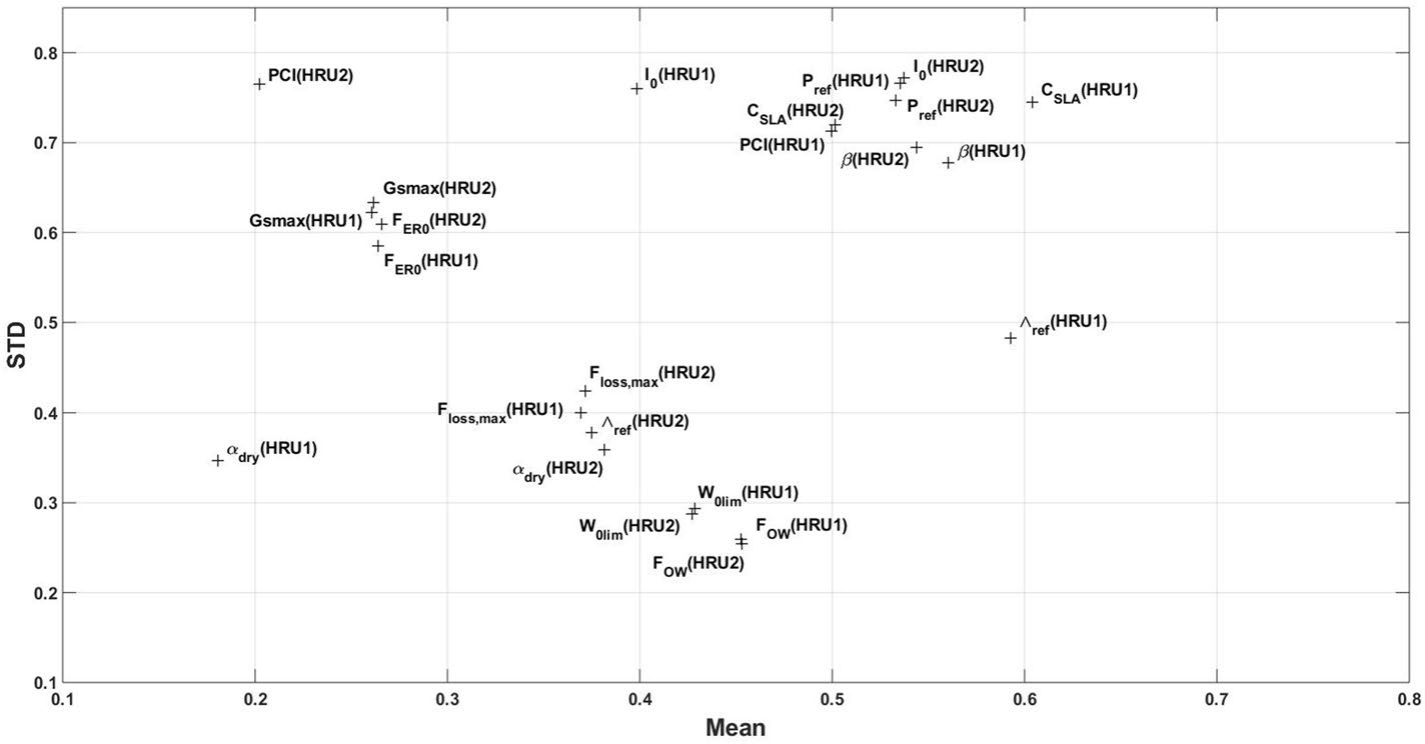
The scatter plot of average and STD of parameters’ weights from the sensitivity analysis.

In addition to the above variations, it is found that the sensitivity of parameters shows considerable variations over different grid points. This can be seen in Fig. 2, where the relationship between the average and STD of parameters over the grid points is shown. These variations indicate that defining fixed values (spatially and temporally) for parameters is not realistic as it does not reflect the characteristics of different regions (and over different time periods) and can be problematic. The large STD values for a majority of parameters such as $I_0$, $C_{SLA}$, $P_{ref}$, and $\beta$ can be explained by larger spatial variabilities of the parameters. This is also the case for some parameters with smaller weights, e.g., *PCI* (in HRU2 corresponding to short and shallow-rooted vegetation). It can also be seen that parameters with larger variabilities such as $C_{SLA}$, $\Lambda _{ref}$, $P_{ref}$, $\beta$, and $I_{0}$ demonstrate larger sensitivities too (cf. Fig. 1). This means that the model is largely sensitive to the variations of these parameters. A few parameters such as ${\alpha }_{dry}$, ${F}_{loss,max}$, and $W_{0lim}$, on the other hand, show smaller spatial variabilities and can be considered spatially homogeneous. Based on this test, we focus only on the most sensitive and variable parameters including $C_{SLA}$, $\Lambda _{ref}$, $I_{0}$ $P_{ref}$, and $\beta$ to be estimated. This allows to efficiently improve the model by avoiding estimation of all parameters.

### Parameter estimation

**Figure 3:**
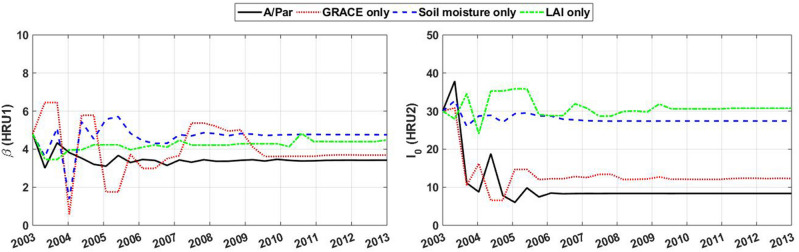
Average parameter values for $\beta$ and $I_0$ over the Mississippi basin during the assimilation period derived by different data assimilation cases.

The parameter estimation results are presented here. The adjusted parameters and their range of variations from the application of the assimilation approach are presented in Table 2. Figure 3 shows the time evolution of two sample parameters ($\beta$ and $I_0$) over the assimilation period. The variation of these two parameters represents their average at each month for the Mississippi basin. From the figure, it can be seen that the parameter estimation process converges the parameters for different assimilation cases, i.e. GRACE, soil moisture, LAI only experiments, as well as simultaneous data assimilation. Details of the converged parameters over both basins can be found in Table 2. The results are for both multivariate and univariate data assimilation scenarios. The STD values show the spread of parameters around the average value, which indicates the variabilities and corresponding uncertainties of the estimated parameters. Table 2 shows that some parameters have larger STDs, e.g., $\beta$, $I_0$, ${\Lambda }_{ref}$, $P_{ref}$, $C_{SLA}$, which generally suggests more spatial variability. These results suggest the ability of the parameter estimation approach to derive different values for parameters by adequately spanning the parameter space. Spatially varying parameters can better capture the characteristics of areas with different atmospheric and environmental conditions. Moreover, it is found that the estimated parameters are considerably different from the initial values, especially for the A/Par approach, which will consequently affect state estimates too. It can also be inferred from the table that each assimilation scenario results in different parameter estimation. Nevertheless, closer results can be found between the multivariate case (A/Par) and GRACE-only assimilation. This can be explained the larger impact of the GRACE data during the assimilation process compared to the other assimilated observation. This will be investigated more in the following section. Furthermore, to better explore the corresponding impact of the parameter estimation on model simulations, the simulations with (A/Par) and without (A/O) the adjusted parameters are analyzed (cf. “Results validation–Observations impact” sections).

**Table 2:** Estimated parameters for the multivariate and univariate data assimilation experiments.

	$\beta$	$I_0$	${\Lambda }_{ref}$	$P_{ref}$	$C_{SLA}$
A/Par					
HRU1	$3.35\pm 1.42$	$12.78\pm 7.48$	$2.24\pm 1.14$	$[4.79\pm 3.55] \times 1\hbox{e}+5$	$44.18\pm 12.63$
HRU2	$8.16 \pm 4.59$	$8.51 \pm 5.08$	$1.92 \pm 0.56$	$[4.46 \pm 4.90] \times 1\hbox{e}+5$	$52.21 \pm 15.18$
GRACE only					
HRU1	$3.86 \pm 5.56$	$15.17 \pm 7.93$	$2.58 \pm 1.46$	$[2.29 \pm 2.15] \times 1\hbox{e}+5$	$36.22 \pm 16.51$
HRU2	$6.20 \pm 7.81$	$12.86 \pm 5.45$	$1.50 \pm 0.40$	$[3.14 \pm 3.73] \times 1\hbox{e}+5$	$47.13 \pm 12.95$
Soil moisture only					
HRU1	$4.84 \pm 4.29$	$27.82 \pm 4.14$	$2.43 \pm 0.94$	$[3.07 \pm 5.29] \times 1\hbox{e}+3$	$16.48 \pm 6.16$
HRU2	$5.39 \pm 6.77$	$28.49 \pm 2.38$	$1.61 \pm 0.21$	$[3.79 \pm 5.72] \times 1\hbox{e}+3$	$21.34 \pm 5.82$
LAI only					
HRU1	$4.51 \pm 4.47$	$29.44 \pm 3.18$	$2.17 \pm 0.73$	$[2.80 \pm 3.69] \times 1\hbox{e}+3$	$24.07 \pm 11.75$
HRU2	$4.75 \pm 6.35$	$31.08 \pm 2.76$	$1.77 \pm 0.19$	$[2.72 \pm 3.31] \times 1\hbox{e}+3$	$31.65 \pm 9.37$

Each parameter is represented using its average values and its uncertainty at the 95% confidence interval. HRU1 and HRU2 represents deep-rooted (tall) and shallow-rooted (short) vegetation, respectively.

### Results validation

**Figure 4:**
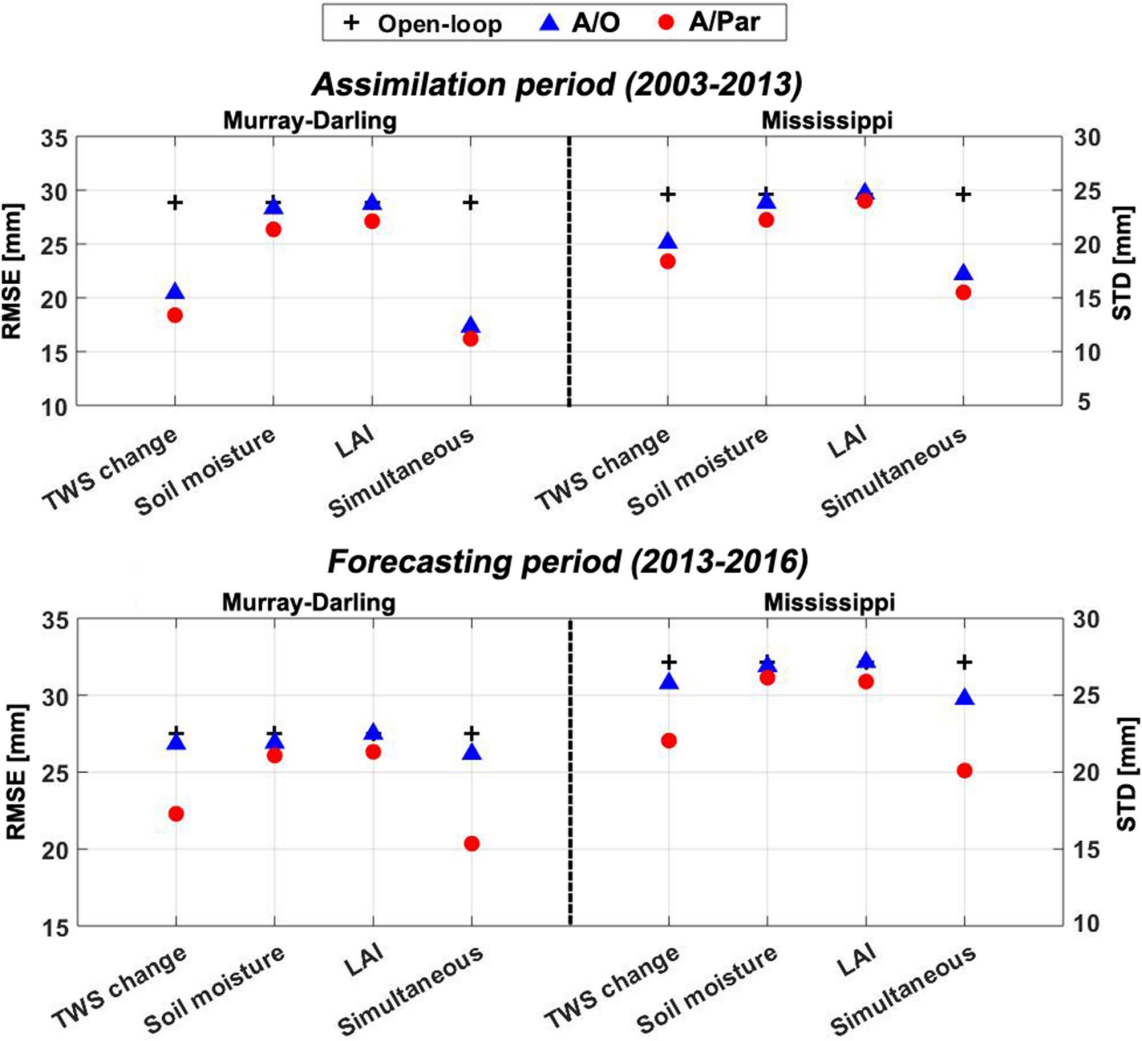
Average groundwater RMSE and STD computed for each method (A/O and A/Par) and multivariate (simultaneous assimilation) and univariate (GRACE TWS only, satellite soil moisture only, and LAI only) data assimilation using groundwater in-situ measurements for assimilation and forecasting periods.

**Figure 5:**
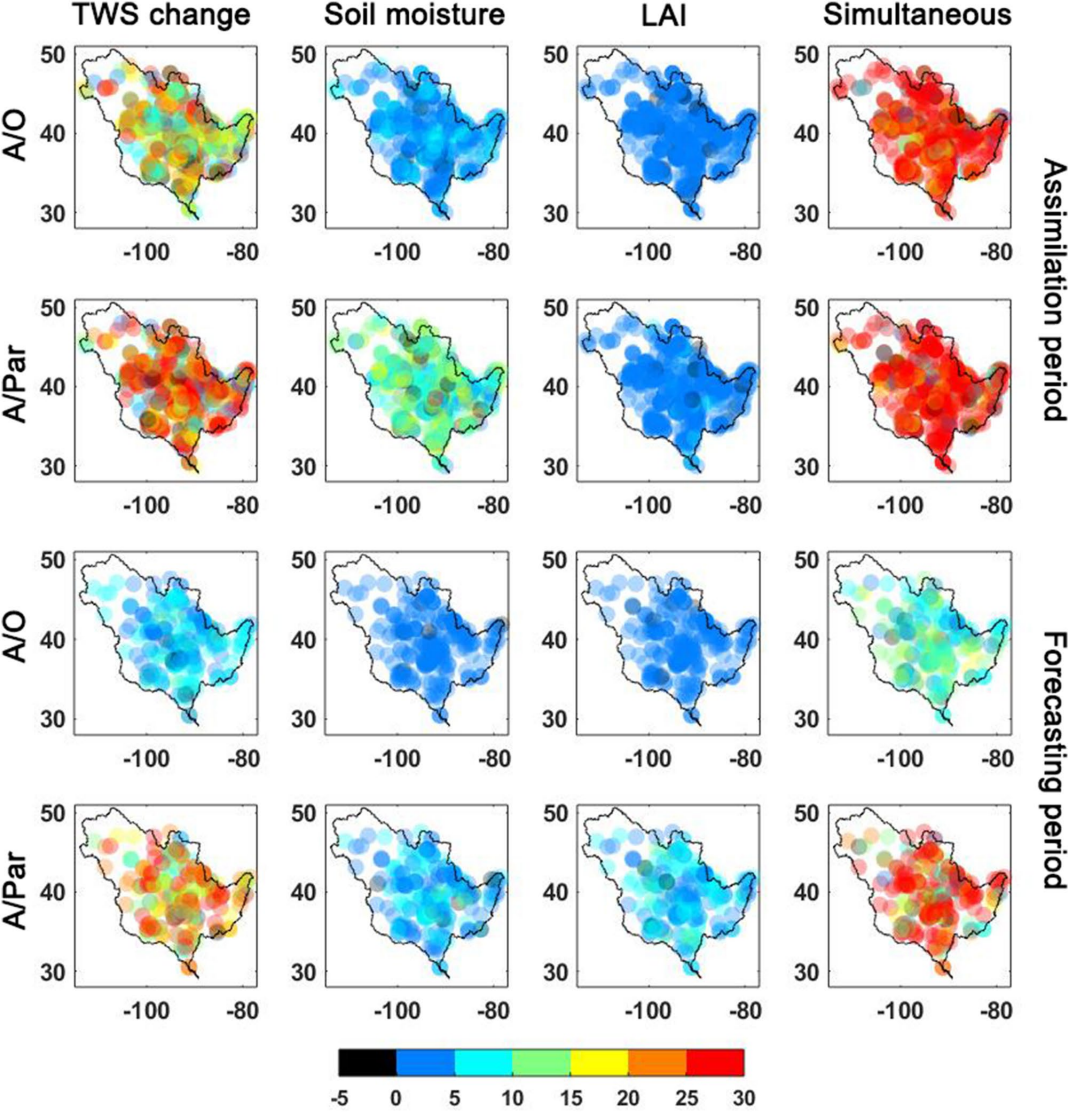
Percentage groundwater RMSE reduction for each assimilation case for A/O and A/Par over the Mississippi basin within the assimilation and forecasting periods.

Independent in-situ measurements over the Murray–Darling and Mississippi basins are also used to evaluate the results for A/O and A/Par approaches. We compare the results of assimilating different observations, i.e. GRACE TWS only, satellite soil moisture only, LAI only, and simultaneous assimilation of all three data products. To this end, RMSE and STD values for both the assimilation and forecasting periods are computed (Fig. 4). We further compare RMSE values for groundwater wells and the different assimilation methods both for the assimilation and forecasting periods (Fig. 5). The figure shows the RMSE reduction for each scenario with respect to the open-loop results. Overall, the results highlight the effectiveness of the satellite data assimilation for improving the model simulations, especially over the assimilation period. Moreover, multivariate data assimilation clearly achieves the best results over both basins. This can clearly be seen for different locations in Fig. 5. Multivariate data assimilation performs reasonably consistent across the basin for both experiment periods. GRACE data assimilation reduces RMSE and STD more than soil moisture and LAI only assimilation experiments. This is expected due to the larger impact of GRACE TWS on groundwater storage during assimilation. Despite this, it is observed that simultaneous (multivariate) A/Par reduces groundwater RMSE 32% (on average) compared to the open-loop run, which is the best performance amongst the different assimilation cases. Similar performance can be observed for the two basins. The A/Par method also obtains slightly better results compared to the A/O method over the assimilation period. Over the forecasting period, however, the multivariate simultaneous data assimilation method performs substantially better, which is evident from smaller RMSE values in both basins compared to the open-loop and A/O results. This can also be seen in Fig. 5, where simultaneous data assimilation (and to a lesser degree also GRACE only assimilation using A/Par) results in higher RMSE reductions than A/O. Such superiority can be explained by the positive impacts of the new method on model parameters, which allows the model to preserve the adjustment impact during the forecast period.

**Figure 6:**
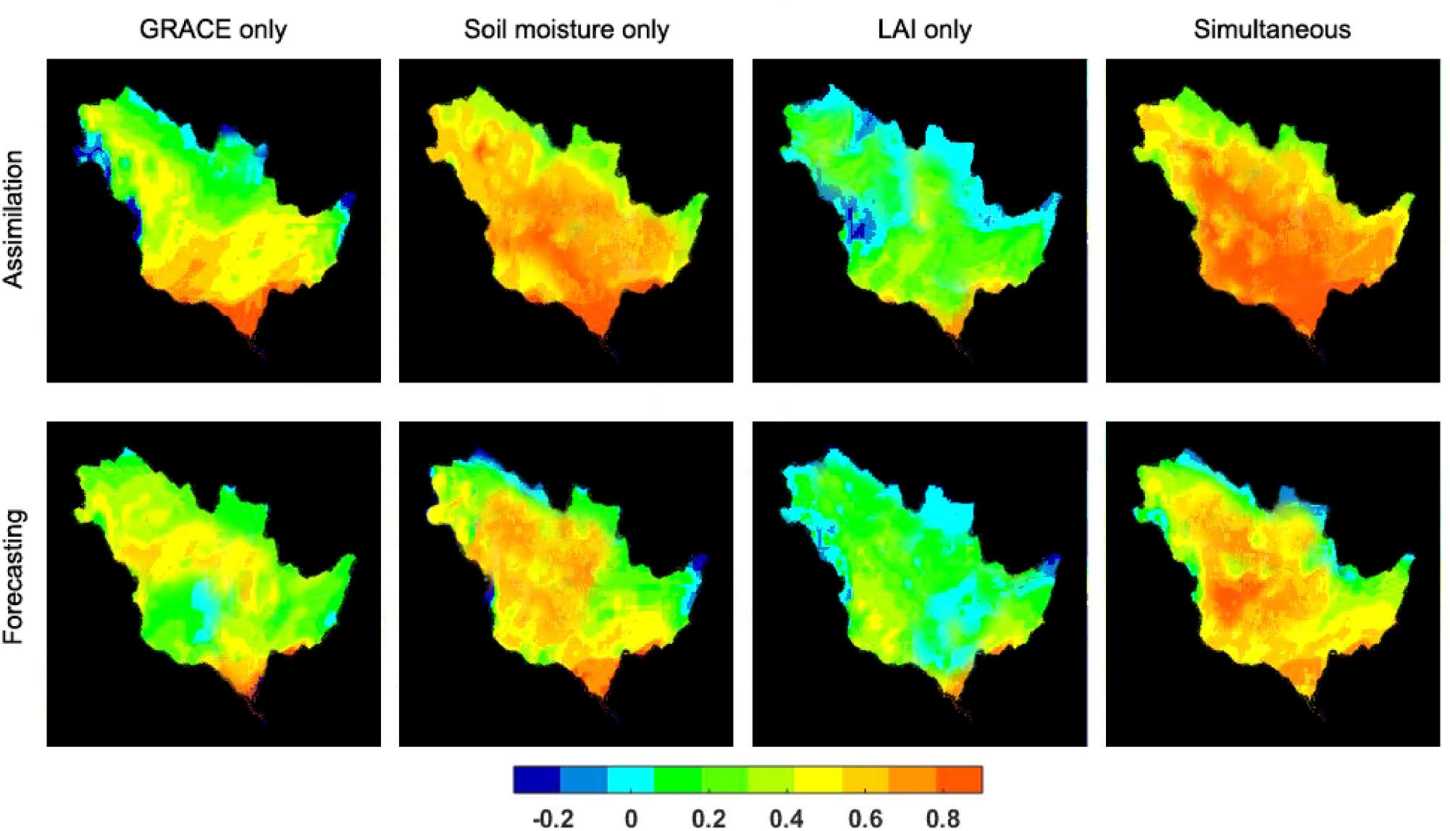
Soil moisture correlation improvement from the open-loop simulation to different cases of data assimilation with respect to ASCAT observations.

The performance of the above data assimilation scenarios is further assessed against in-situ and independent satellite soil moisture measurements relying only on the correlation analysis. Correlations between simulated soil moisture (with and without data assimilation using different observations) and in situ measurements are calculated at different depths and average results are reported in Table 3. For this purpose, the top layer estimates are examined against in-situ measurements of 0–8 cm for Murray–Darling and 0–10 cm for Mississippi. The estimated top, shallow and a portion of deep-root soil layers are compared with in-situ measurements of deeper layers over the two basins (e.g., 0–30 cm and 0–90 cm for Murray–Darling, and 0–50 cm and 0–100 cm for Mississippi). A statistical test is also applied to measure the significance of the results at 0.05 level. In general, assimilating multiple observations simultaneously leads to higher correlation values, both for the A/Par and A/O methods compared to the open-loop results. Furthermore, top layer simulated soil moisture is compared with the surface soil moisture L2 product from the Advanced SCATterometer (ASCAT) over the same periods. The ASCAT soil moisture products provide an estimate of the water saturation of the 5 cm topsoil layer and are derived from the European Organisation for the Exploitation of Meteorological Satellites (EUMETSAT). The correlation between the open-loop soil moisture and the soil moisture of the data assimilation scenarios with ASCAT soil moisture data is then calculated to derive improvement values, i.e. the difference in correlation for a data assimilation approach and the open loop experiment, both for the assimilation and forecasting period (Fig. 6).

**Table 3:** Average correlation values calculated from in-situ measurements and soil moisture estimates from for open-loop simulations, data assimilation runs using different observations, and data assimilation with and without parameter estimation.

Method	Mississippi basin		Murray–Darling basin	
	Assimilation period	Forecasting period	Assimilation period	Forecasting period
Open-loop	0.63	0.61	0.71	0.74
A/O				
GRACE TWS	0.67	0.61	0.75	0.74
Soil moisture	0.75	0.63	0.81	0.75
LAI	0.65	0.60	0.75	0.74
Simultaneous	0.83	0.64	0.87	0.76
A/Par				
GRACE TWS	0.69	0.64	0.78	0.76
Soil moisture	0.77	0.72	0.83	0.79
LAI	0.65	0.63	0.73	0.74
Simultaneous	0.85	0.79	0.88	0.82

According to Table 3, the multivariate data assimilation improves correlation values by 0.21 (on average for the cases with and without parameter estimation) over the Mississippi basin and by 0.17 (on average for the cases with and without parameter estimation) over the Murray–Darling basin. It can be seen that univariate satellite soil moisture data assimilation performs the best among the univariate data assimilation experiments by increasing the correlation values from 0.67 (on average for open-loop) to 0.76. Similar results can also be seen in Fig. 6, where the multivariate data assimilation obtains the highest correlation improvement followed by soil moisture only data assimilation. Limited impacts can be observed by GRACE data assimilation, especially over the assimilation period while the LAI only assimilation case has no considerable impact on the results. From Table 3, improvements can also be seen in soil moisture estimates from GRACE data assimilation. Overall, it is found that GRACE only data assimilation mainly affects the deep-root and shallow soil zones within the assimilation period (on average $\sim 9\%$ more than top layer) while soil moisture data assimilation largely improves top layer estimates (on average $\sim 12\%$ more than deep-root layer). The former can be explained by the larger impact of GRACE TWS data assimilation (as in uni- and multivariate cases) on deeper model soil layers. Satellite soil moisture measurements, on the other hand, mainly reflect the top few centimeter soil water variations and correspondingly impact the model top layer. The combination of observations in the simultaneous case leads to the better performance of the approach in both A/O and A/Par. Between the experiment periods, more correlation improvement (with respect to the open-loop results) is obtained during the forecasting period using A/Par ($\sim 20\%$ for simultaneous assimilation) than A/O ($\sim 4\%$). This shows the importance of multi-mission observations during data assimilation. Yet, estimating parameters along with the state effectively improves the state-parameter estimates when multivariate data assimilation is assumed. This effect can also be observed in Fig. 6. The simultaneous data assimilation, and to a lesser degree soil moisture only scenario positively impacts the model top layer simulation by estimating parameters along with states during the assimilation period.

**Figure 7:**
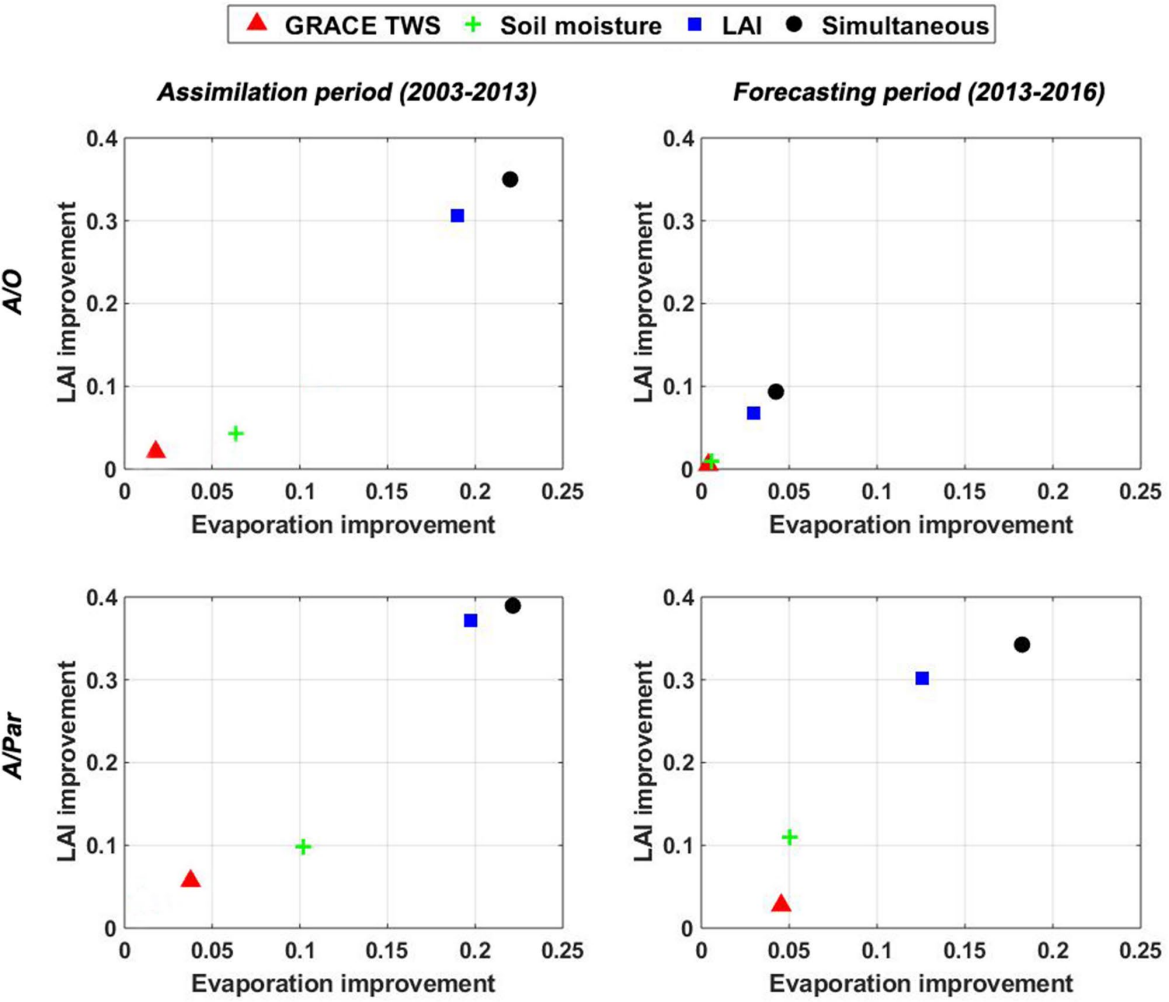
Correlation improvement in LAI and evaporation estimates from different assimilation cases (with and without parameter estimation for univariate and multivariate observations) with respect to AVHRR LAI and MOD16 observations compared to the open-loop results (averaged) over the Murray–Darling and Mississippi basins for the assimilation (2003–2013) and forecasting (2013–2016) periods.

Further result evaluation is done to assess the effect of satellite data assimilation, specifically from the LAI products. As shown in literature^90–92^, constraining land surface models with LAI observations could result in better evapotranspiration predictions. To explore this, the estimated LAI and evapotranspiration by the A/O and A/Par approach are compared with AVHRR LAI and evapotranspiration from the MODIS Global Evapotranspiration Project (MOD16)^93^. This is done also for all univariate and multivariate assimilation cases. Average correlation improvement with respect to the open-loop results for the Mississippi and Murray–Darling basins are depicted in Fig. 7. The analysis is done again separately for the assimilation and forecast periods. One can see that data assimilation effectively improves the estimates in most of the cases. The improvements are more pronounced for simultaneous and LAI only data assimilation. GRACE only and soil moisture only data assimilation cases lead to a small level of correlation enhancement, especially using the A/Par method, which can be explained by the updated parameters. Improvements in LAI simulations clearly lead to evapotranspiration estimates closer to MOD16. The improvements are found for both the assimilation with and without parameter estimation approaches, particularly over the assimilation period. The best results over the forecast period are found for the A/Par experiments and for simultaneous data assimilation. The A/O performance is clearly worse compared to the A/Par performance over the forecast period. These results are consistent with the previous assessments, stressing that multi-satellite data assimilation, especially along with parameter estimation considerably improves the model simulations by incorporating various observations. Due to the superiority of the multivariate data assimilation cases based on this section’s results, in the following, we focus only on these approaches and especially A/Par to investigate their performance in more aspects.

### Observations impact

**Figure 8:**
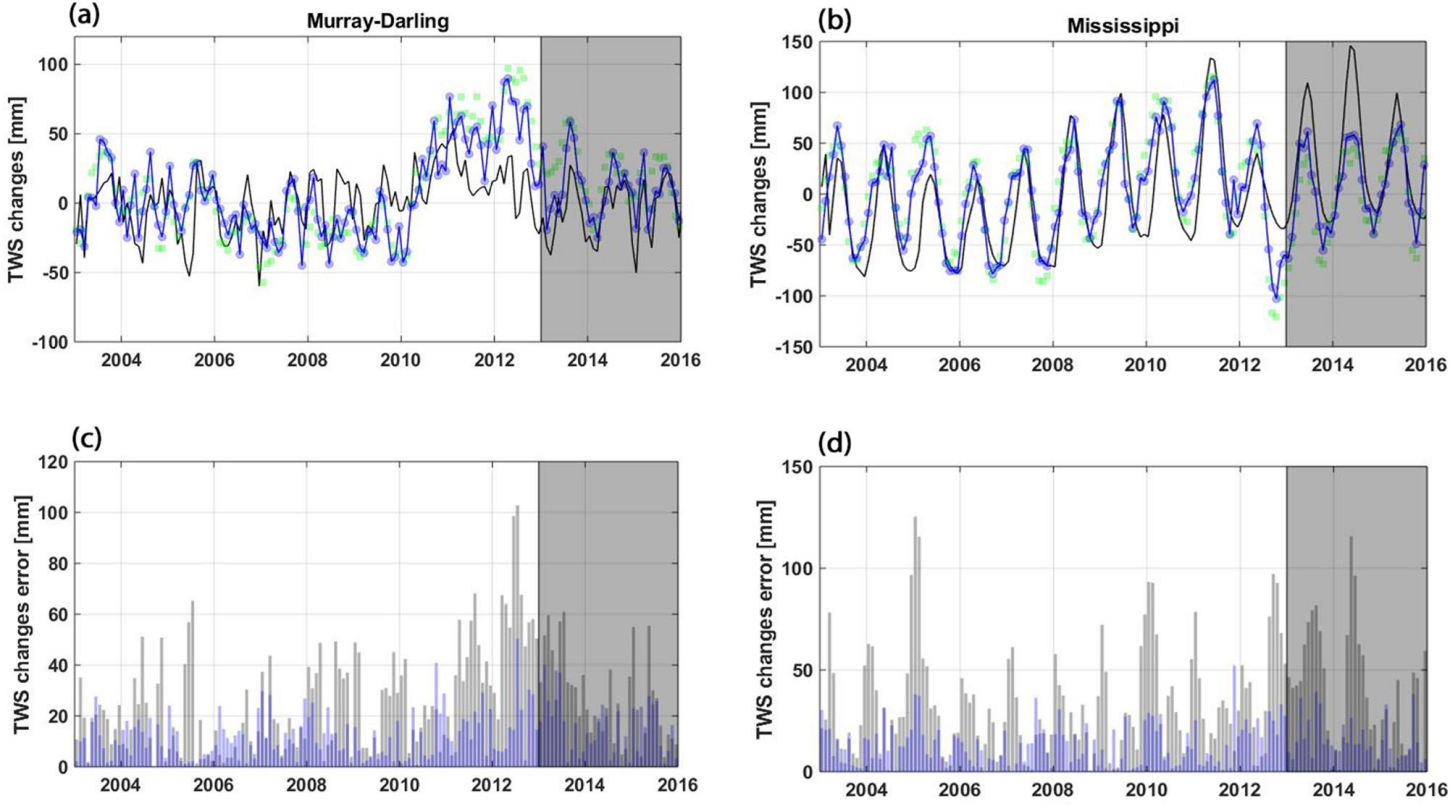
(**a**) and (**b**) show average TWS variation with (blue) and without (black) data assimilation, as well as GRACE TWS (green) over Murray–Darling and Mississippi basins, respectively. The corresponding errors, i.e., the difference between GRACE TWS and those from each method are presented in (**c**) and (**d**). Note that the solid vertical line at 2003 separates data assimilation and forecast periods.

The integration of multivariate satellite observations (GRACE TWS, soil moisture, and LAI simultaneously) during the assimilation process impacts model simulations. This effect can be seen in Fig. 8 over the Mississippi and Murray–Darling basins. In this figure, basin-averaged TWS variations from the open-loop run (no data assimilation) are compared with the assimilation results, as well as the GRACE TWS data. The error, measured as the absolute difference between the GRACE TWS data and model simulations (with and without assimilation) is also plotted in Fig. 8c,d. Note that the forecast period (2013–2016), when no assimilation is applied, is separated from the assimilation period (2003–2013). It can clearly be seen in Fig. 8 that data assimilation decreases misfits between the open-loop and observations over both basins. Smaller errors in Fig. 8c,d confirm the ability of the applied data assimilation method for decreasing discrepancies between the model and observations. This improvement can largely be seen for the assimilation period, and to a lesser degree, for the forecast period. Importantly, data assimilation leads to a better simulation of anomalies such as 2011–2012 over the Murray–Darling basin and 2012–2013 over the Mississippi basin.

**Figure 9:**
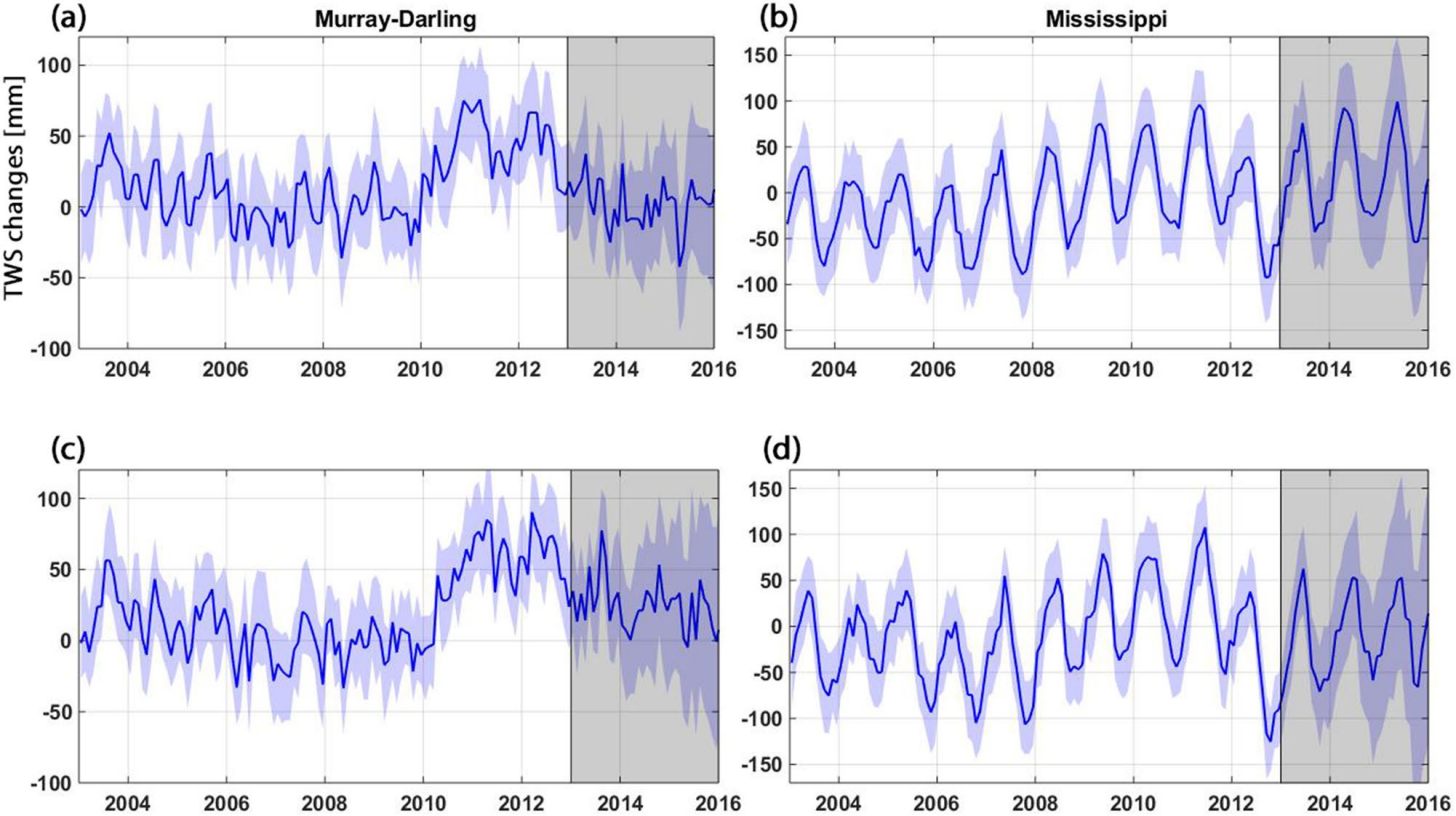
Ensemble TWS variations for the A/Par method over Murray–Darling (**a**) and Mississippi (**b**) basins. (**c**) and (**d**) demonstrate ensemble TWS variations for the A/O method over Murray–Darling and Mississippi basins, respectively. Solid blue lines show the average ensemble.

To further investigate the impact of observations in the assimilation process, TWS ensemble spread over the basins is shown in Fig. 9. This is particularly of interest to monitor the influence of data assimilation on estimates in the assimilation period and its absence in the forecast period. TWS variations from individual ensemble members (shaded blue) and their average (solid blue) are displayed in Fig. 9. To better explore the effect, the comparison is done between the A/Par (Fig. 9a,b) and A/O (Fig. 9c,d) approaches. Both methods maintain the ensemble spread steadily during the assimilation period. While the pattern for both methods is similar over the assimilation period they differ in the forecast period. Larger spreads and corresponding uncertainties can be observed for the A/O results compared to the A/Par approach (cf. Fig. 9c,d). It can be inferred from the figure that the parameter estimation process along with the assimilation can extend the impact of data assimilation during the forecast period. This also reduces model uncertainties in that time period.

**Figure 10:**
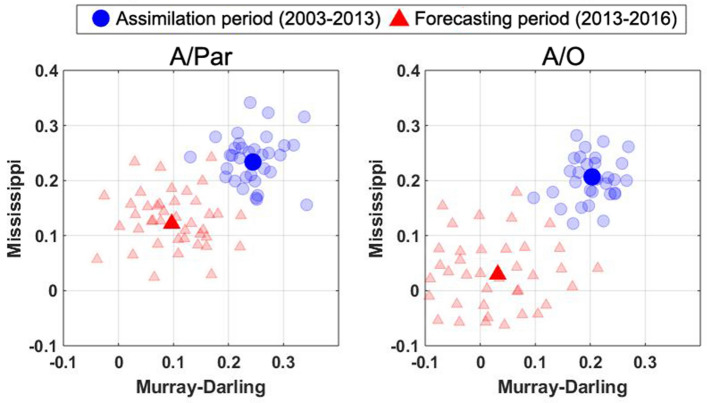
Soil moisture correlation improvement by assimilation methods, i.e. against the open-loop run with respect to the satellite observations over the assimilation and forecasting periods. Each circle and triangle belong to separate ensemble members.

Figure 10 shows the impact of data assimilation on soil moisture components from individual ensemble members to further investigate the simulation results (cf. Fig. 9). The correlation improvements over both basins are calculated with respect to the open-loop run, i.e., $r_c-r_o$ with $r_c$ being the correlation coefficients between the assimilation (A/Par and A/O) results and satellite soil moisture observations and $r_o$ being the correlation coefficients between the open-loop results and satellite soil moisture observations. This is done separately for the assimilation and forecast periods. Figure 10 depicts correlation increases by both assimilation methods over the assimilation period. The A/Par method, however, obtains slightly better results, especially over the Mississippi basin, with an average increase of correlation of 0.29 compared to 0.25 for A/O. Over the forecast period, on the other hand, the new method performs remarkably better than the A/O approach over both basins, which is related to the estimated parameters. In addition, it can be seen from the figure that ensemble correlations show a larger spread over the forecast period, particularly for the A/O approach. This can indicate the larger stability of the A/Par method during the forecast period (as Fig. 9), which can result in smaller model state uncertainties.

**Figure 11:**
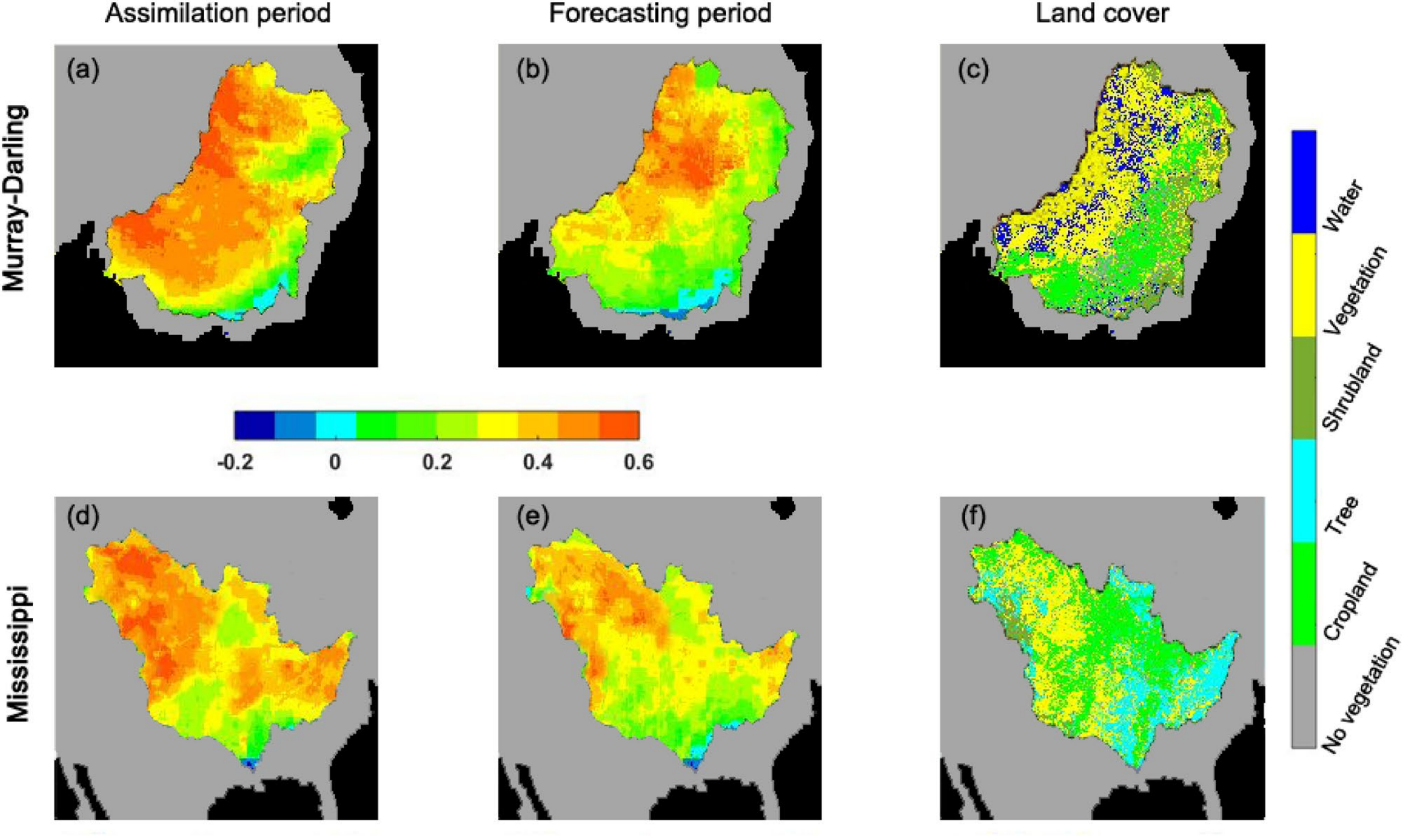
LAI correlation improvement for the A/Par approach. The correlation values are calculated with respect to the assimilated LAI data for both the assimilation and open-loop results. The difference is then computed as the correlation improvement for the assimilation period over the Murray–Darling (**a**) and Mississippi (**d**) basins, as well as for the forecasting period over the Murray–Darling (**b**) and Mississippi (**e**) basins. Land cover data (sampled for 2013) is presented in (**c**) for Murray–Darling basin and (**f**) for Mississippi basin.

Now we explore the influence of the assimilated LAI data products on estimates. To this end, we compare the estimated LAI from two assimilation approaches by comparing it with LAI derived from AVHRR data. This is again explored over the Murray–Darling and Mississippi basins. Figure 11 shows the correlation improvement with respect to the open-loop run. Correlation values are computed for the assimilation period over each grid point. Land cover data acquired from Climate Change Initiative - European Space Agency (Version 2.0; http://www.esa-landcover-cci.org/) is also presented in the figure for a better interpretation of LAI improvement results. From Fig. 11, the A/Par method increases the correlation compared to the open-loop results over both basins.The correlation improvement over the forecast period, however, is smaller, i.e. more than 0.4 improvement over $\sim$31% of grid points (averaged over both basins) against $\sim$74% for the assimilation period. This is expected due to the absence of data assimilation. Nevertheless, correlation increase can be seen across the basins within the forecast period. More improvements can be seen over the vegetated areas (containing trees, vegetation, and shrubland) in both assimilation and forecasting periods compared to the cropland areas. This can be attributed to the higher capability of the assimilated data to reflect the variations of plant canopies. Overall, it can be concluded that the method successfully incorporates observations during the filtering process and estimates the associated parameters. This is in correspondence with the previous findings as documented in Figs. 8, 9 and 10.

### Evaluation against water fluxes

**Figure 12:**
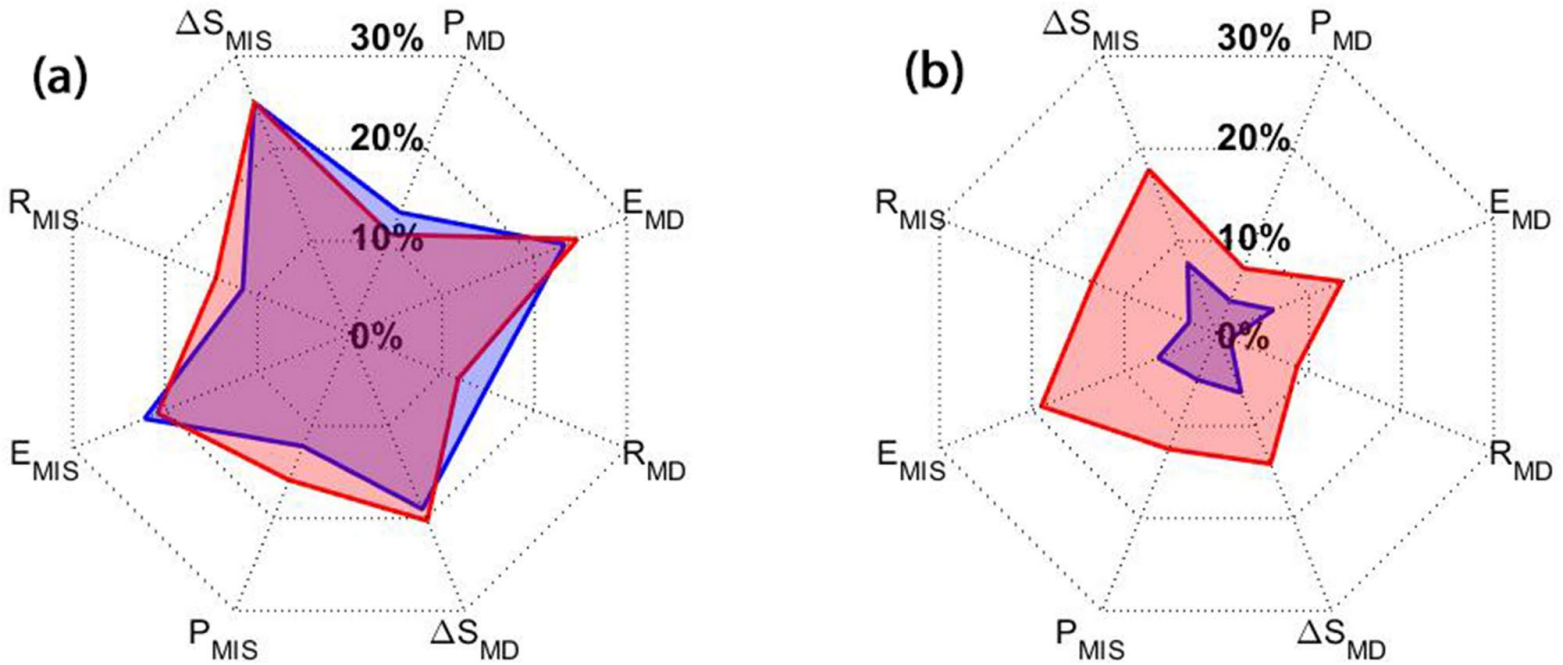
Average cross correlation increase between the estimated TWS and various flux observations within the Murray–Darling (with MD subscript) and Mississippi (with MIS subscript) basins. (**a**) presents the results over the assimilation period and (**b**) shows the results over the forecasting period. Red and blue graphs display the A/Par and A/O results, respectively.

A successful data assimilation approach for a water balance system not only improves the model simulations of various compartments but should also result in a better reproduction of water fluxes. To assess this, the updated TWS estimates are compared against flux observations of precipitation, evaporation, water discharge, and water storage changes using correlation analysis. Cross-correlation values are computed between the simulations (from the open-loop run, as well as assimilation with and without parameter estimation) and flux observations used in the second step of data assimilation filtering scheme (cf. “Model and data” section). Afterwards, improvement is calculated between the assimilation results with respect to the open-loop results for the assimilation (Fig. 12a) and forecasting (Fig. 12b) periods, separately for the Murray–Darling (indicated by ‘MD’) and Mississippi (indicated by ‘MIS’) basins. Both assimilation methods improve the agreement between measured and modelled flux components and storage over the assimilation period. The cross-correations increase stronger for evapotranspiration and water storage changes, which can be explained by the assimilation of TWS and LAI data from satellite products. The level of improvement over the forecasting period is much better for the A/Par approach than for the A/O approach. This can be seen clearly in Fig. 12b, where the A/Par results are approximately 12% (on average) better than those of A/O. This shows that the applied parameter estimation strategy has more pronounced impacts than A/O on results.

### Climate variabilities

**Figure 13:**
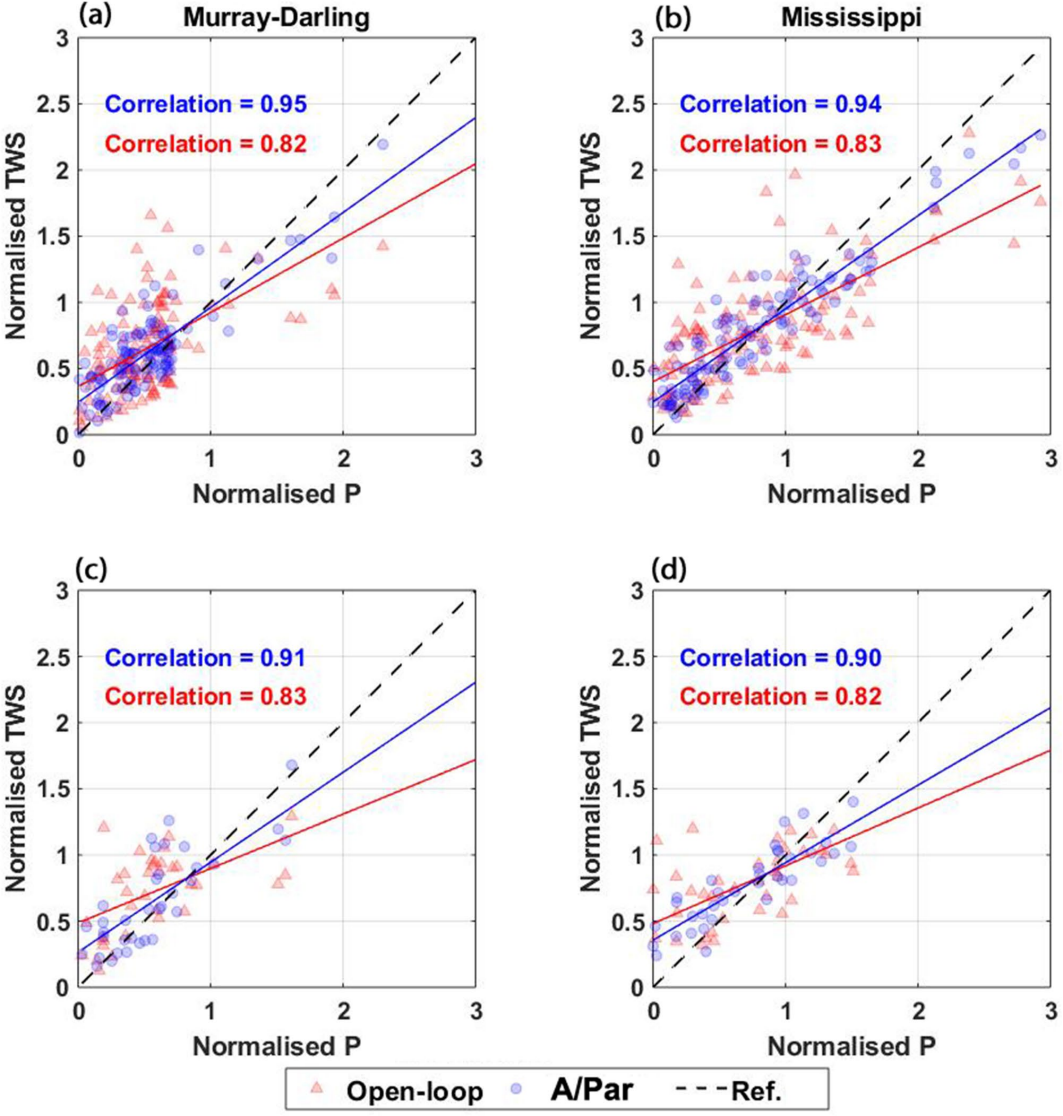
Scatter plots of TWS estimates and precipitation (P) for the assimilation (top panel) and forecasting (bottom panel) periods. Solid blue and red lines represent the values for the assimilation and open-loop results. The black dashed line indicates the reference values.

In this section, the ability of the multivariate data assimilation with parameter estimation technique (as the best method so far) to accurately reflect inter-annual weather variabilities as well as extreme events is assessed. Figure 13 plots average TWS variations from the open-loop and A/Par approach with respect to precipitation data over the Murray–Darling and Mississippi basins. This is done separately for the assimilation and forecasting periods. Better agreement between the two time series leads to a higher correlation between precipitation and TWS anomalies. The assimilation results show a better match than the open-loop results between the estimated TWS-variations and precipitation variations. This is clearer over the assimilation period, in which the A/Par method increases the correlation by 0.12 (on average) compared to the open-loop simulations. Improvement can also be found over the forecasting period over both basins (by 0.08) using the multivariate A/Par approach. These results demonstrate that the assimilation results better represent climate-induced variations compared to the open-loop run.

**Figure 14:**
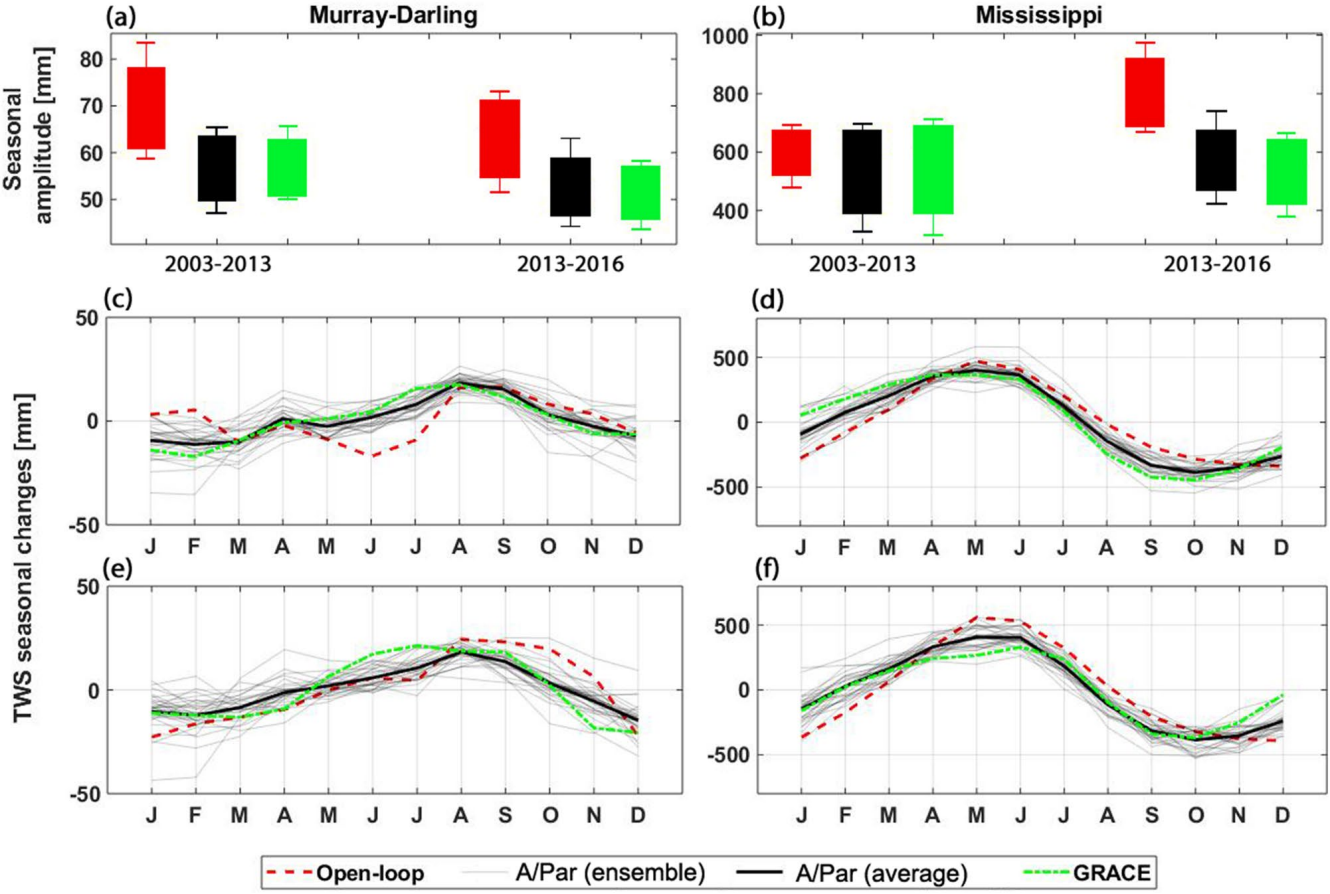
TWS seasonal amplitude from A/Par (black), open-loop (red), and GRACE (green) in (**a**) (Murray–Darling) and (**b**) (Mississippi). Corresponding TWS seasonal changes are shown in (**c**) and (**d**) for the assimilation period and (**e**) and (**f**) for the forecasting period.

Another important aspect of successful model simulations is their ability to represent seasonal changes. This is evaluated by comparing seasonal variations of the open-loop and A/Par TWS results with those from GRACE data (Fig. 14). Results in Fig. 14 depict the average TWS seasonal amplitude (top panel) and TWS seasonal changes (middle and bottom panels) for the Murray–Darling and Mississippi basins over the assimilation and forecasting periods. Figure 14 illustrates that contrary to the open-loop result, assimilation results show not only similar seasonal amplitude but also closer range of variations compared to GRACE. Importantly, such an improvement can also be observed over the forecasting period (2013–2016), which is related to the estimated model parameters by remote sensing data assimilation.

Better agreement between the assimilation results and observations can also be seen in seasonal changes over both study periods. This is more evident for the Murray–Darling basin, where larger discrepancies exist between the open-loop results and GRACE data. Data assimilation, thus, has larger impacts in this case even over the forecasting period. It can be concluded that the assimilation results agree better to climatic variations due to their better performance in representing seasonal changes, which are triggered largely by climate-related components mainly through precipitation.

**Figure 15:**
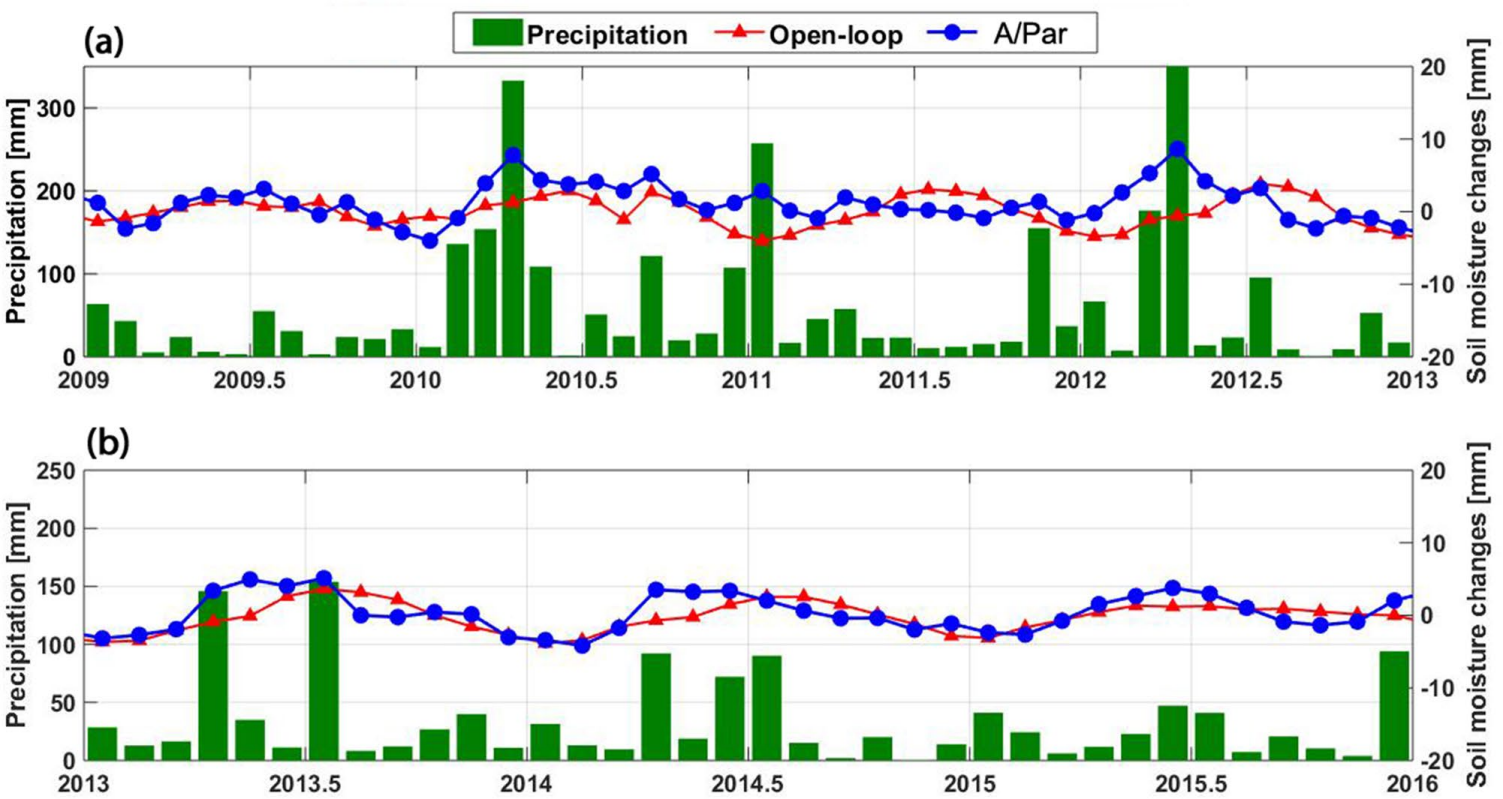
Average soil moisture changes from the open-loop and A/Par, as well as precipitation over the Murray–Darling basin for the periods of 2009–2013 (**a**) and 2013–2016 (**b**).

To further investigate the performance of data assimilation, soil moisture results are compared with average precipitation changes over two particular time periods, 2009–2013 (in assimilation period) and 2013–2016 (forecasting period) for the Murray–Darling basin. The former time period is selected due to the occurrence of an extreme (or irregular) climatic event namely high precipitation due to El Niño Southern Oscillation between 2010 and 2012^94^. The latter time period is selected to monitor the assimilation impacts on the forecastings. Figure 15 shows the top layer soil moisture variations from the A/Par method and the open-loop run, as well as precipitation variations. Based on the figure, the assimilation results give anomalies that better match the corresponding anomalies in precipitation data than the open-loop results. This can, for example, be found in 2010 and 2012 over the assimilation period. The poor performance of the open-loop results compared to the assimilation outcomes can be due to various factors such as erroneous model parameters, over-simplifying physical phenomena, and errors in its underlying equations. Better results for the A/Par approach suggest that estimating parameters through data assimilation can largely address the issue and consequently reflect anomalies. A similar performance can also be seen over the forecasting period, e.g., when positive anomaly in 2013 is clearer in the A/Par results. This again confirms the positive impact of A/Par on the parameter estimations.

**Figure 16:**
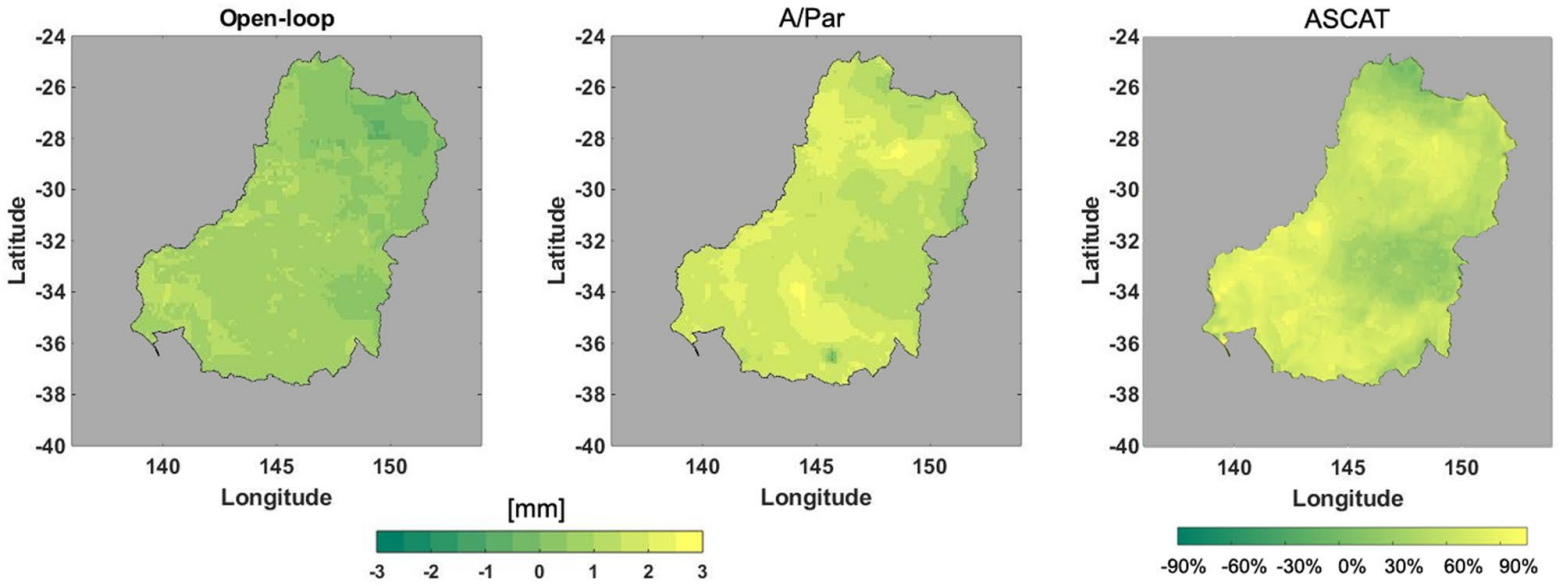
Spatial changes of soil moisture from March–April 2010 to January–February 2010 over the Murray–Darling basin for the open-loop, A/Par, and ASCAT observations.

To better illustrate this, the difference between average soil moisture content in March–April and January–February 2010 over the Murray–Darling basin is shown in Fig. 16, again both for the A/Par method and the open-loop run. This is done to investigate the impact of ENSO phenomena on soil moisture changes. Remarkably larger positive differences in the assimilation results indicate their better performance in representing the phenomena. These results show that assimilating multiple satellite data products can effectively improve the model skills to capture inter-annual weather anomalies.

## Conclusions

The present study investigated the ability of multivariate satellite remote sensing data assimilation to improve predictions with a land surface model. Various observations including GRACE TWS, AMSR-E and SMOS soil moisture products, and AVHRR LAI were assimilated individually and simultaneously into the W3RA model using the recently proposed A/Par method, UWCEnKF. This was done for (i) state-parameter estimation over the assimilation period and (ii) for model predictions over the forecasting period. Different data sets were used to assess the data assimilation performance over the Murray–Darling and Mississippi basins. The major findings of this effort are:

- In general, it was shown that the application of multi-mission satellite data can successfully improve the model’s different estimates, both in the assimilation and forecasting periods. On the other hand, univariate data assimilation was found to mainly improve the model corresponding variable. Analysing the results against the assimilated observations shows that the A/Par method results in a closer correspondence to observation data, including independent, not assimilated, data. Thus, this study showed the importance of multivariate data assimilation when various water components are targeted combined with parameter estimation.
- In the forecasting period, the joint assimilation and parameter estimation method still improves estimates considerably, but the A/O approach does not improve updates. Better TWS and LAI forecasts were obtained over the Murray–Darling and Mississippi basins by this method. The use of independent groundwater and soil moisture measurements also confirmed this. The UWCEnKF A/Par method demonstrated high capability to preserve the observations’ impacts over a longer time period, which suggests that the method can successfully estimate the model parameters. Furthermore, multivariate assimilation along with parameter estimation shows promising performance in reflecting inter-annual weather variabilities as well as weather extremes into the state estimates over both assimilation and forecasting periods. Therefore, model parameter estimation during data assimilation is crucial for improved predictions.

Overall, based on both assessments against assimilated and independent observations, multivariate data assimilation with model parameter estimation remarkably improved model simulations, e.g., in terms of water storage accuracy and forecasting skill. Nevertheless, more investigation is required on the performance of the method on hyper-resolution models, where assimilating massive datasets can be problematic. Moreover, the method should be tested over various basins with different hydro-climatic conditions to further assess its impact on the simulations, especially for the forecasting periods.
